# Maternal gut microbiota mediates prenatal stress-induced fetal blood‒brain barrier dysfunction

**DOI:** 10.1080/19490976.2026.2631242

**Published:** 2026-02-19

**Authors:** Xuanping Wang, Fang-Yue Zhou, Ting Wu, Chenchi Duan, Xukai Luo, Yicong Meng, He-Feng Huang, Yan-Ting Wu

**Affiliations:** a The International Peace Maternity and Child Health Hospital, Shanghai Key Laboratory of Embryo Original Disease, School of Medicine, Shanghai Jiao Tong University, Shanghai, China; b Institute of Reproduction and Development, Shanghai Key Laboratory of Reproduction and Development, Obstetrics and Gynecology Hospital, Fudan University, Shanghai, China; c Institute of Medical Genetics and Development, Key Laboratory of Reproductive Genetics (Ministry of Education) and Women's Hospital, Zhejiang University School of Medicine, Zhejiang, China; d Shanghai Key Laboratory of Female Reproductive Endocrine Related Diseases, Shanghai, China

**Keywords:** Prenatal stress, blood–brain barrier, gut microbiota, interferon β pathway, tryptophan

## Abstract

Maternal prenatal stress confers elevated neuropsychiatric risk to offspring, yet the mechanisms underlying fetal neurodevelopmental impairment remain elusive. The gut microbiota has emerged as a key regulator of brain development and behavior. However, the mechanisms mediating the interactions between the microbiota and the developing brain are still poorly understood. Here, utilizing a prenatal stress mouse model integrated with multi-omics approaches, comprehensive behavioral assays, and molecular validations, we demonstrate that prenatal stress not only induces maternal gut microbiota dysbiosis during pregnancy but also, more critically, leads to fetal blood‒brain barrier (BBB) developmental defects and subsequent abnormalities in emotional behavior and cognitive function in adult offspring. Maternal probiotic supplementation during gestation can reverse both gut microbial dysbiosis and fetal BBB dysfunction. Notably, transcriptomic analysis reveals that the maternal gut microbiota modulates interferon-β (IFN-β) signaling along the placenta‒fetal brain axis under stress. Furthermore, metabolomic profiling suggests that prenatal stress exposure profoundly influences the maternal fecal and serum metabolome. In conclusion, our findings establish a placenta‒brain axis wherein maternal microbial signals orchestrate fetal neurovascular development, identifying microbiota-targeted interventions as a neuroprotective strategy.

## Introduction

The intrauterine environment critically shapes fetal development and influences health outcomes across the entire lifespan. During the prenatal period, the brain undergoes rapid growth, rendering it particularly vulnerable to adverse exposures. Recent years have witnessed growing recognition of the importance of maternal mental health during pregnancy. Notably, the conceptualization of gestational psychological well-being has evolved from a singular focus on postpartum depression to encompass a broader spectrum of conditions, including depression, anxiety, and stress. Stress is a nonspecific response to a threat or noxious stimuli with resultant damaging consequences. Preclinical models have demonstrated that prenatal stress induces depression- and anxiety-like behaviors in pregnant dams, which are associated with detrimental outcomes in offspring, including increased susceptibility to neurological disorders such as anxiety, depression, and communicative disorders.^1^^,^^2^

Accumulating evidence suggests an association between altered BBB permeability and psychiatric disorders.^3^^,^^4^ The structural and functional integrity of the BBB is primarily maintained by endothelial cells interconnected through tight junctions (TJs) and adherens junctions (AJs).^5^ TJs represent specialized transmembrane complexes composed of claudin family proteins, occludins, and junctional adhesion molecules (JAMs), which are associated with cytosolic adapter proteins such as ZO-1 and ZO-2. BBB disruption can precede or exacerbate the progression of neurodegenerative and neurological disorders.^6-8^ Accumulating evidence also indicates altered neurovascular function in stress-related and psychiatric conditions.^3^ Chronic social stress disrupts BBB integrity, promoting depression-like behaviors in female mice.^9^ Therefore, we sought to investigate whether maternal stress during gestation impairs fetal BBB development in offspring.

Previous studies support the gut microbiota as a key regulator of neurodevelopment, with germ-free (GF) models demonstrating lifelong BBB permeability when maternal microbial signaling is absent.^10^ Crucially, the gestational window of BBB maturation (E15.5–18.5 in mice) coincides with peak vulnerability to environmental insults, establishing a plausible mechanistic link between prenatal stress and neurovascular dysfunction.

To address these gaps, the present study employed a prenatal restraint stress model combined with behavioral assays, molecular profiling, morphological analysis, transcriptomics, metabolomics and 16S rRNA sequencing to investigate offspring BBB alterations under maternal stress conditions and elucidate the underlying mechanisms. The primary objectives of this study were to demonstrate that prenatal stress induced fetal BBB developmental defects and establish the maternal gut microbiota as the critical mediator of this effect. Furthermore, we investigated whether probiotic treatment could restore BBB integrity in stressed offspring by ameliorating maternal gut dysbiosis. Finally, we analyzed the molecular pathways through which the maternal gut microbiota influences fetal BBB development under stress conditions.

## Methods

### Animals model

Timed mating was performed to obtain embryos at defined time points after conception. Females with vaginal plugs were determined as pregnant at embryonic day 0.5 (E0.5). Pregnant mice were randomly assigned to distinct experimental cohorts using a computer-generated randomization protocol to ensure unbiased group allocation. The number of dams in each group was at least 6. The stress protocol followed the previously described method,^11^ employing a 7-d gestational stress paradigm for model establishment. The stress group dams were subjected to daily restraint stress in ventilated 50 mL conical tubes for 2 h at a fixed time between E11.5 and E17.5, while control group dams remained undisturbed. At E18.5, behavioral tests were conducted on all groups of pregnant dams. Following behavioral assessment, a subset of dams from each group was sacrificed for tissue collection, while the remaining dams were allowed to deliver offspring for subsequent behavioral testing during adulthood. For adult offspring experiments, pups were delivered naturally and housed with their biological dams. All animals were housed under a 12 h light/12 h dark cycle and held under specific pathogen-free conditions. To ensure statistical validity and avoid pseudo-replication (litter effects), only one male and/or one female offspring from each litter was selected for any specific experiment (e.g., behavioral tests or RNA-seq). Each experimental group consisted of offspring derived from at least 6–8 distinct dams. The animal protocol was reviewed and approved by the Experimental Animal Welfare Ethics Committee of the International Peace Maternity and Child Health Hospital affiliated with Shanghai Jiao Tong University. Pregnant mice were deeply anesthetized with 4% isoflurane.

### Probiotic treatment

This study employed a standardized dosing protocol based on published literature^12^ using the composite probiotic formulation Lacidofil® (Lallemand Health Solutions, Montreal, Canada) containing live bacterial strains: *Lactobacillus rhamnosus* R0011 (95%) and *Lactobacillus helveticus* R0052 (5%). The experimental procedure consisted of: (1) reconstituting the lyophilized powder in sterile distilled water to a final concentration of 1 × 10^9^ CFU/mL; (2) administering via ad libitum drinking water from the vaginal plug detection until gestational day 18.5 (or prior to parturition); and (3) refreshing the bacterial solution every 48 h to maintain probiotic viability. Based on routine monitoring, the average daily water consumption per dam was approximately 8–10 mL.

### Cell culture and transfection

Brain microvascular vessel-derived endothelial cells (bEnd.3) were cultured in Dulbecco's modified Eagle's medium (DMEM) supplemented with 10% FBS (GIBCO) and maintained at 37 °C in a humidified atmosphere containing 5% CO_2_. The medium was refreshed every 48 h. 3 × 10^5^ cells were seeded on 6-well plates. Upon reaching 30%–50% confluence, the cells were transfected with mouse *Ifnar1* siRNA (sense: CACGGUCGCUGUAGAAGUATT, Obio Technology, Shanghai, China) using Lipofectamine RNAiMAX reagent (Thermo Fisher Scientific). Twenty-four hours post-transfection, the cells were treated with or without 1-methyl-D-tryptophan (1-MT, 1 mM) for 12 h. Transfection efficiency was evaluated 36 h after transfection via real-time PCR according to the manufacturer's recommendations.

### Behavioral assays

At E18.5, dams from different groups underwent the open field test to assess anxiety-related behaviors. For offspring, male and female mice at six weeks-old were subjected to behavioral tests in the following order: the open field test (OFT), novel object recognition (NOR), three-chambered social approach task (3-CST), elevated zero maze (EZM), and tail suspension test (TST). A minimum 48-h interval was maintained between consecutive behavioral tests. All the behavioral experiments were conducted under acoustically controlled conditions to minimize external disturbances. The objects and cages were cleaned with a 75% ethanol solution to remove any odor cues. All videos were recorded and analyzed by using the TopScan system

#### Open field test

The OFT was used to assess anxiety-like behavior. The mice were placed in a 50 × 50 cm square enclosure with 40 cm high walls and allowed to explore freely for 5 min. The time spent in the central zone and the distance covered by the animal were calculated. The investigator was blinded to the identity of the animals throughout the experiments.

#### Novel object recognition

The NOR test serves as a classical behavioral paradigm for assessing cognitive dysfunction.^13^ The experimental procedure consists of three phases: during the habituation phase, the mice are allowed to freely explore an empty square open field (50 × 50 × 40 cm) for 10 min; in the familiarization phase, the mice explore two identical objects for 10 min before being returned to their home cages for a 1-h interval (during which one object is replaced with a novel object); and finally, during the test phase, the mice's 10-min exploratory behavior toward both familiar and novel objects is recorded (defined as sniffing or touching the objects with vibrissae or forepaws). The recognition index is calculated according to established methods.^14^

#### Three-chambered social approach task

The three-chamber social test was performed to assess sociability and preference for social novelty.^15^ Mice were tested in the three-chamber apparatus following a 30 min period of habituation in the testing room. For the sociability test, an unfamiliar mouse (strain- and sex-matched) was introduced into a wire cup in one side chamber, serving as the social stimulus, while an empty cup served as the control in the other. The doors were opened, and the subject was allowed to explore the entire apparatus for 10 min. Immediately following the sociability phase, the preference for social novelty test was conducted. The original stranger mouse (stranger 1) remained in its enclosure (serving as the “familiar” mouse), while a novel unfamiliar mouse (stranger 2) was placed in the previously empty wire cup. The subject mouse was again allowed to freely explore all three chambers for another 10 min. The time spent in each chamber and the time spent sniffing the wire cups were recorded automatically throughout both phases.

#### Elevated zero maze

The exploration activity was measured to assess anxiety-related behaviors. The testing apparatus was elevated 75 cm above floor level under standardized fluorescent illumination. During the 5-min testing session, the mice were permitted unrestricted exploration of the arena. The behavioral parameters quantified included the time spent in the open and closed arms and the distance traveled in the open and closed arms.

#### Tail suspension test

The TST was measured to assess depression-related behaviors. The tip of the mouse tail was fixed with medical tape, and the mice were hung on a shelf 30 cm above the cage floor. A 6 min test session was videotaped and scored by a trained observer who was blinded to the experimental conditions. The total immobility duration of the mice during the final 4 min of the test was analyzed.

### Molecular analyses

#### Western blotting

Total protein lysate (30 μg per lane) was loaded on 4%–20% Bis–Tris polyacrylamide gels (Epizyme Biotech). SDS‒PAGE was run at 140 V for 1 h. Proteins were transferred to PVDF membranes by fast transfer buffer for 30–45 min at 400 mA. The membranes were blocked in 5% non-fat milk for 120 min at room temperature and probed with the following antibodies at 4 °C: Claudin-5 (1:1000, Thermo Fisher Scientific, 352500), Occludin (1:500, Thermo Fisher Scientific, 404700), ZO-1 (1:1000, Thermo Fisher Scientific, 617300), and beta-actin (1:1000, Cell Signaling Technology, 4967S). Then the membranes were incubated with appropriate horseradish peroxidase-conjugated secondary antibodies for 1 h at room temperature after being washed in TBST for 3 × 10 min. Signals were detected using an Amersham ImageQuant™ 800 detection system (Cytiva) following the manufacturer's recommendations.

#### Quantitative PCR

Total RNA was extracted with the RNAeasy kit according to the manufacturer's instructions (Beyotime). The RNA concentration and purity were assessed using spectrophotometric analyses of 260/280 ratios. RNA was reverse transcribed using the high-capacity cDNA reverse transcription kit (Thermo Scientific), amplified using SYBR Green Master Mix (Yeasen), and detected by QuantStudio (TM) 7 Flex System (Applied Biosystems). All Ct values were obtained in triplicate. The primer sequences are listed in Table 1. The analysis of output values was made using standard ΔΔCt method with beta-actin as an endogenous control.

**Table 1.** List of murine expression primers used in this study.

Gene	Forward​	Reverse​
Actin​	GTGACGTTGACATCCGTAAAGAC​	CTAGGAGCCAGAGCAGTAATCTC​
Claudin5​	GCAAGGTGTATGAATCTGTGCT​	GTCAAGGTAACAAAGAGTGCCA​
Occludin​	TTGAAAGTCCACCTCCTTACAGA​	CCGGATAAAAAGAGTACGCTGG​
ZO-1​	CTGGTGAAGTCTCGGAAAAATG	CATCTCTTGCTGCCAAACTATC​
TLR4​	CAGCAGAGGAGAAAGCAT​	CACCAGGAATAAAGTCTCTG​
Zonulin​	GCACTTGGTTCGCTATCGCT​	GCTTCTCGTCGTTTAAGGTGTA​
IL-6​	TAGTCCTTCCTACCCCAATTTCC​	TTGGTCCTTAGCCACTCCTTC​
IL-1β​	GTCTTCCTAAAGTATGGGCTG​	CACAGGCTCTCTTTGAAC​
TNF-α​	TGTGCTCAGAGCTTTCAACAA​	CTTGATGGTGGTGCATGAGA​
Ifnar1​	AGCCACGGAGAGTCAATGG​	GCTCTGACACGAAACTGTGTTTT​
Irf7​	CTTCAGCACTTTCTTCCGAGA​	TGTAGTGTGGTGACCCTTGC​
Ifit1​	CAGAAGCACACATTGAAGAA​	TGTAAGTAGCCAGAGGAAGG​
Ifit3​	GCCGTTACAGGGAAATACTGG​	CCTCAACATCGGGGCTCT​
Oas1a​	TGTCCTGGGTCATGTTAATAC​	CCGTGAAGCAGGTAGAGA​
Gbp6​	GAAGGAAGCTGGAGCAGGA​	TCTCAGTTGCTGTATCTCTTTGTT​
Iigp1​	GAGCCTGTAGCAGTGAAGGT​	GCTGACCCATGACTTCAAGC​

Note: all primers were obtained from BGI Genomics, Shanghai, China.

### Enzyme-linked immunosorbent assay

Blood samples were collected by the submandibular bleeding method and allowed to clot at room temperature for 2 h, followed by centrifugation at 1000 × *g* for 20 min. The serum supernatant was collected, aliquoted, and stored at −80 °C for subsequent use. Corticosterone levels in maternal serum were measured using a competitive enzyme-linked immunosorbent assay and following the manufacturer's protocol (E-EL-0161c, Elabscience).

### Hematoxylin–eosin staining (H&E)

Intestinal tissues were collected and fixed in 4% PFA (Biosharp) for 24 h. The fixed tissues were dehydrated through a graded ethanol series, cleared in xylene, and embedded in paraffin for sectioning at a thickness of 4 μm. After the paraffin sections were dewaxed and hydrated, the nuclei were stained with hematoxylin solution (Solarbio), and then, cytoplasmic staining was performed with eosin staining solution (Solarbio). After the slices were dried, the sheets were preserved with neutral resin (SCR).

### Immunofluorescence staining

Tissues were fixed with 4% paraformaldehyde (PFA) overnight. Immunofluorescence was performed on brain coronal cryotome sections blocked with bovine serum albumin (BSA) (Solarbio), followed by incubation with primary antibody as follows: mouse anti-occludin (1:400, Invitrogen, 331500), mouse anti–Claudin-5 (1:400, Invitrogen, 352500), rabbit anti-laminin (1:50, Sigma, L9393), rabbit anti-Iba1 (1:300, Wako, 019-19741) and rabbit anti-cFos (1:400, CST, 2250). Then, the sections were washed with PBS (3 × 5-min washes) and incubated with secondary goat anti-mouse Alexa 488- or Cy3-labeled goat anti-rabbit IgG (H+L). Then sections were counterstained with DAPI for 10 min to visualize the cell nuclei. Slides were visualized using a fluorescence microscope (Nikon Eclipse C1).

### Immunohistochemistry

Samples were fixed in 4% PFA (Biosharp) overnight, dehydrated through an ethanol gradient series, and embedded in paraffin. For fetal brain tissues, coronal sections were prepared using an RM 2016 microtome (Leica), mounted on glass slides, rehydrated, and treated with 20 mM citrate buffer (pH 6.0) for 30 min. Endogenous peroxidase activity was blocked with 3% hydrogen peroxide solution, followed by 30 min of blocking nonspecific epitopes with 3% bovine serum albumin (Solarbio) at room temperature.

The following primary antibodies were used for labeling fetal brain and intestinal tissues: IL-6 (ab6672, Abcam), IL-1β (12242, CST), and TLR4 (ab22048, Abcam). All the primary antibodies were incubated with the tissue sections at 4 °C overnight. After washing, the sections were incubated with secondary antibodies for 50 min. Diaminobenzidine (DAB) was used as the chromogen for visualization, followed by counterstaining with hematoxylin (Solarbio). Finally, the slides were examined under an XSP-C204 microscope (CIC).

### BBB and neurovascular assessment

#### Embryonic BBB permeability assay

The experimental protocol was established based on prior studies.^14^ Fetal mice were obtained via cesarean section from anesthetized dams. A 10-kDa dextran-tetramethylrhodamine (4 mg/mL, D3312, Invitrogen) was administered via a Hamilton syringe through precise intrahepatic injection (10 μL) in embryos while maintaining intact umbilical circulation to minimize hemodynamic perturbations. Following equivalent circulatory distribution, whole fetal brains were carefully dissected and collected. Embryonic heads were fixed by immersion in 4% PFA overnight at 4 °C. The fixed tissue was embedded in OCT (Sakura) and frozen sections were taken. The sections were blocked with 3% BSA and washed in PBS. Then, the slices were immunostained with Laminin primary antibody (1:50, L9393, Sigma) overnight at 4 °C, followed by secondary goat anti-rabbit Alexa 488 for 1 h at room temperature. Images were visualized and acquired using a fluorescence microscope (Nikon Eclipse C1).

#### Transmission electron microscopy

The brains were removed, and the areas of interest were dissected and fixed in 2.5% glutaraldehyde (Sigma) overnight at 4 °C. The specimens were rinsed in 0.1 M PB, pH 7.4, postfixed in 2% osmium tetroxide 0.1 M PB, pH 7.4, at 20 °C for 2 h, dehydrated in ethanol (SCR) followed by acetone and embedded in Pon-812 (SPI). Ultrathin sections (approximately 60–80 nm) were cut by a Leica EM UC 7 (Leica), and performed using a transmission electron microscopy (HT7700, Hitachi).

### Omics analyses

#### RNA-sequencing (RNA-seq) processing and analysis

Total RNA was extracted using the Total RNA Extraction Kit 2.0 Plus (following the manufacturer's protocol). RNA purity and concentration were measured using a NanoDrop 2000 spectrophotometer (Thermo Scientific, USA). RNA libraries were sequenced on the Illumina NovaSeq 6000 platform at OE Biotech Co., Ltd. (Shanghai, China). The raw FASTQ data underwent quality control using FastQC (v0.11.9), followed by low-quality read filtering with FASTP (v0.20.1).^16^ Clean reads were aligned to the mouse reference genome (NCBI_GRCm39) using HISAT2.^17^ Differential expression analysis was performed with DESeq2, and hierarchical clustering of the differentially expressed genes was conducted using R software (v3.2.0). Significantly enriched terms were identified through GO enrichment analysis, while Gene Set Enrichment Analysis (GSEA) was performed using GSEA software.^18^

#### Microbial community analysis

Fecal samples were collected from each experimental group at E18.5, followed by microbial genomic DNA extraction. Subsequent library preparation and sequencing were performed by Majorbio Bio-Pharm Technology Co., Ltd. (Shanghai, China). Briefly, total microbial genomic DNA was extracted and amplified using specific primer pairs targeting the V3–V4 hypervariable regions. The purified PCR products were used to construct sequencing libraries, which were then subjected to paired-end sequencing on an Illumina Nextseq2000 platform (Illumina, USA). The raw sequencing data underwent quality control using fastp (v0.19.6)^19^ and sequence assembly with FLASH (v1.2.11). Processed reads were analyzed through the QIIME2 pipeline with DADA2 plugin for denoising, generating amplicon sequence variants (ASVs). Taxonomic classification of ASVs was performed using the Naive Bayes classifier in QIIME2 with reference to the SILVA 138 16S rRNA database (v138). Rarefaction curves and alpha diversity indices (including observed ASVs, Chao1 richness index, Shannon diversity index, and Good's coverage) were calculated using Mothur (v1.30.1).^20^ Microbial community dissimilarities among samples were assessed by principal component analysis (PCA) and principal coordinate analysis (PCoA). The linear discriminant analysis effect size (LEfSe) method was employed to identify significantly differentially abundant bacterial taxa (from the phylum to the genus level) across the experimental groups.^21^

#### Metabolomics analysis

Maternal serum and fecal samples were collected at E18.5 from each experimental group for untargeted metabolomics analysis. For serum metabolomics, 100 μL aliquots of serum were transferred to 1.5 mL centrifuge tubes and mixed with extraction solvent. After vortex mixing, samples underwent low-temperature ultrasonication for 30 min, followed by protein precipitation at −20 °C. Reconstitution was performed with 100 μL solution (acetonitrile:water = 1:1, followed by 5-min low-temperature ultrasonication and centrifugation to collect the supernatants. For fecal metabolomics, fecal samples were placed in 2 mL centrifuge tubes containing a 6-mm diameter homogenization bead. Metabolites were extracted using a methanol/water solution (4:1, v/v) containing internal standards (e.g., 0.02 mg/mL L-2-chlorophenylalanine). The samples were homogenized for 6 min at 50 Hz (−10 °C), followed by ultrasonic extraction (40 kHz, 5 °C) for 30 min. The samples were subsequently incubated at −20 °C for 30 min and centrifuged at 13,000 × *g* for 15 min at 4 °C. The resulting supernatants were transferred to LC‒MS vials for analysis. LC‒MS/MS analysis was conducted by Majorbio Bio-Pharm Technology Co., Ltd. (Shanghai, China) using a UHPLC-Orbitrap Exploris 240 system equipped with an ACQUITY HSS T3 column (100 × 2.1 mm, 1.8 μm; Waters, USA) and electrospray ionization (ESI) source operating in both positive and negative modes. The raw data were preprocessed with Progenesis QI software (Waters, USA), with peak response intensities normalized by the sum normalization method to generate standardized data matrices. Orthogonal partial least squares–discriminant analysis (OPLS–DA) was performed using the R package “ropls” (v1.6.2), with metabolites showing VIP > 1 and *p* < 0.05 identified as significantly different. Metabolic pathway annotation was performed using the KEGG database (http://www.genome.jp/kegg/).

### Statistical analyses

Statistical tests were performed using GraphPad Prism 9 software. Normality assumptions were evaluated using the Shapiro‒Wilk test, followed by Levene test, to determine if variances were homogeneous. If datasets were not normally distributed, a non-parametric Mann–Whitney or Kruskal‒Wallis test was used for two or three groups, respectively. Student's *t-*test was used to evaluate significant differences between two groups of data. For data involving more than two groups, a one-way analysis of variance (ANOVA) followed by Tukey's multiple comparison test. Two-way ANOVA with Sidak's multiple-comparison test was used for ≥2 groups with two variables. Correlation analysis was performed using the Spearman's rank correlation test. In each figure, *n* denotes the number of independent biological replicates. The results were reported as mean ± standard error of the mean (s.e.m.). Statistical significance was defined as *p < 0.05, **p < 0.01, ***p < 0.001, and ****p < 0.0001.

## Results

### Prenatal stress induces maternal anxiety-like behaviors and gut microbiota dysbiosis

**Figure 1.**
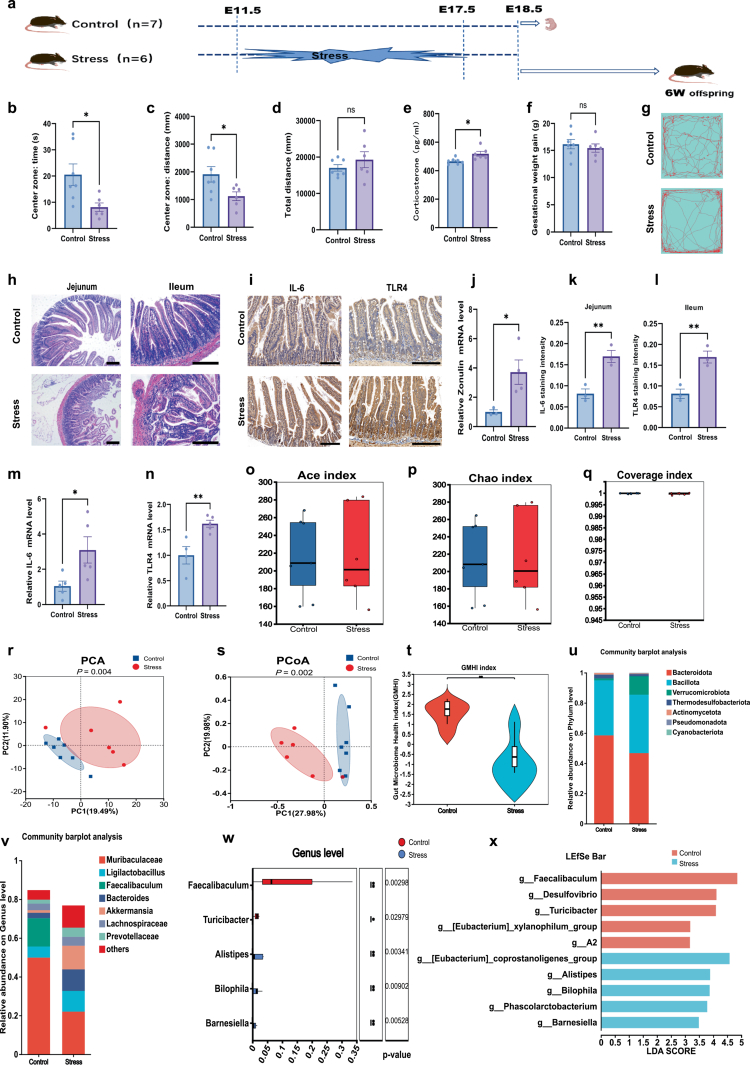
Prenatal stress induces maternal anxiety-like behaviors and gut microbiota dysbiosis. (a) Schematic timeline of experimental procedure. Between E11.5 and E17.5, the stressed group underwent restraint stress for 2 h per day. At E18.5, behavioral tests were conducted on both groups of pregnant dams. Following behavioral assessment, a subset of dams from each group was sacrificed for tissue collection, while the remaining dams were allowed to deliver offspring for subsequent behavioral testing during adulthood. (b and c) Prenatal stress group (*n* = 6) exhibited significantly reduced locomotion time (*p = 0.0223) and distance (*p = 0.0382) in the central zone during the open field test compared to controls (*n* = 7). (d) No significant difference was observed in the total distance traveled between the two groups (*p* = 0.3312). (e) Stress dams (*n* = 6) exhibited significantly elevated serum corticosterone levels (*p = 0.0126) compared to controls (*n* = 7), confirming a sustained stress state. (f) No significant difference in gestational weight gain was observed between the prenatal stress group (*n* = 6) and the control group (*n* = 7). (g) Representative locomotion trajectories of both dam groups in the open field test. (h) HE-stained images of the jejunum and ileum from both dam groups reveal villus atrophy and structural disruption in the stress group. (i) Immunohistochemical staining of IL-6 and TLR4 in intestinal tissues demonstrated stronger positive signals in the stress dams compared to controls. (j) qPCR analysis revealed significant differences in the intestinal gene expression levels of zonulin (*n* = 3 control, 4 stress, and *p = 0.0407) between the two groups. (k) Quantification of IL-6 staining intensity in jejunum sections (*n* = 3 per group, **p = 0.0083). (l) Quantification of TLR4 staining intensity in ileum sections (*n* = 3 per group, **p = 0.0010). (m and n) qPCR analysis revealed significant differences in the intestinal gene expression levels of IL-6 (*n* = 5 per group, *p = 0.0330) and TLR4 (*n* = 4 control, 5 stress, and **p = 0.0081) between the two groups. (o‒q) Wilcoxon rank-sum test bar plot for ACE index (o), Chao index (p) and coverage index (q) from control mice and stress mice at the ASV level. (r and s) Principal component analysis (PCA) and principal co-ordinates analysis (PCoA) of fecal gut microbiota sequencing from control mice or stress mice at the ASV level. (t) Gut microbiome health index (GMHI) based on Shannon index from control mice and stress mice. (u and v) Community bar plot analysis of the gut microbiota sequencing of fecal samples from control mice and stress mice at the phylum level (u) and genus level (v). (w) Wilcoxon rank-sum test bar plot on genus level from control mice and stress mice. (x) LDA effect size (LEfSe) analysis of differentially abundant microbial taxa between control mice (*n* = 7) and stress mice (*n* = 6). Scale bar = 200 μm. The data were presented as mean ± SEM. *p < 0.05, **p < 0.01; ns, and no significant difference. Statistical differences were determined by unpaired two-tailed *t*-tests (b‒f, j‒n).

In the OFT, stressed dams spent significantly less time (*p* = 0.0223, Figure 1b) and traveled shorter distances (*p* = 0.0382, Figure 1c) in the central zone compared to controls, while total locomotion remained comparable (*p* = 0.3312, Figure 1d). Serum corticosterone levels were significantly elevated in the stress group (*p* = 0.0126, Figure 1e), confirming a sustained physiological stress response. Gestational weight gain was unaffected by the stress paradigm (Figure 1f).

Histological analysis of intestinal tissues via hematoxylin and eosin (H&E) staining revealed distinct morphological alterations in stressed dams characterized by villus fragmentation and atrophy (Figure 1h). Consistent with structural impairment, the levels of the tight junction regulator zonulin were significantly elevated in the colon of stressed dams (*p* = 0.0407, Figure 1j). Furthermore, both immunohistochemistry and qPCR analysis demonstrated upregulated protein and mRNA expression of the inflammatory markers IL-6 (*p* = 0.0083 and *p* = 0.0330, respectively; Figure 1k and m) and TLR4 (*p* = 0.0010 and *p* = 0.0081, respectively; Figure 1l and n), indicating a heightened intestinal inflammatory status.

To evaluate the impact of stress on the maternal gut microbiome, we analyzed 16S rRNA gene sequencing data. Examination of alpha-diversity indices—specifically ACE, Chao, and Coverage—revealed no significant differences between the two groups (Figure 1o–q). However, beta diversity analysis via PCA (*p* = 0.004, Figure 1r) and PCoA (*p* = 0.002, Figure 1s) revealed distinct clustering of microbial communities between stressed and control dams. Notably, the gut microbiome health index (GMHI) was significantly lower in the stress group (*p* = 0.0082, Figure 1t), indicating a shift towards a dysbiotic state. These results were further supported by taxa summary analysis at the bacterial phyla and genera levels (Figure 1u and v). Prenatal stress significantly reduced the relative abundance of *Fecalibaculum* (*p* = 0.002, Figure 1w). LEfSe analysis further identified specific microbial signatures: the stress group was enriched in *Bilophila* (LDA score > 3, Figure 1x), whereas the control group showed enrichment of SCFA-producing taxa, including *Fecalibaculum* and *Turicibacter*. Taken collectively, prenatal stress induces anxiety-like behaviors, intestinal barrier impairment, and gut microbiota dysbiosis in maternal mice.

### Prenatal stress induces fetal BBB developmental defects and abnormal behaviors in adult offspring

**Figure 2.**
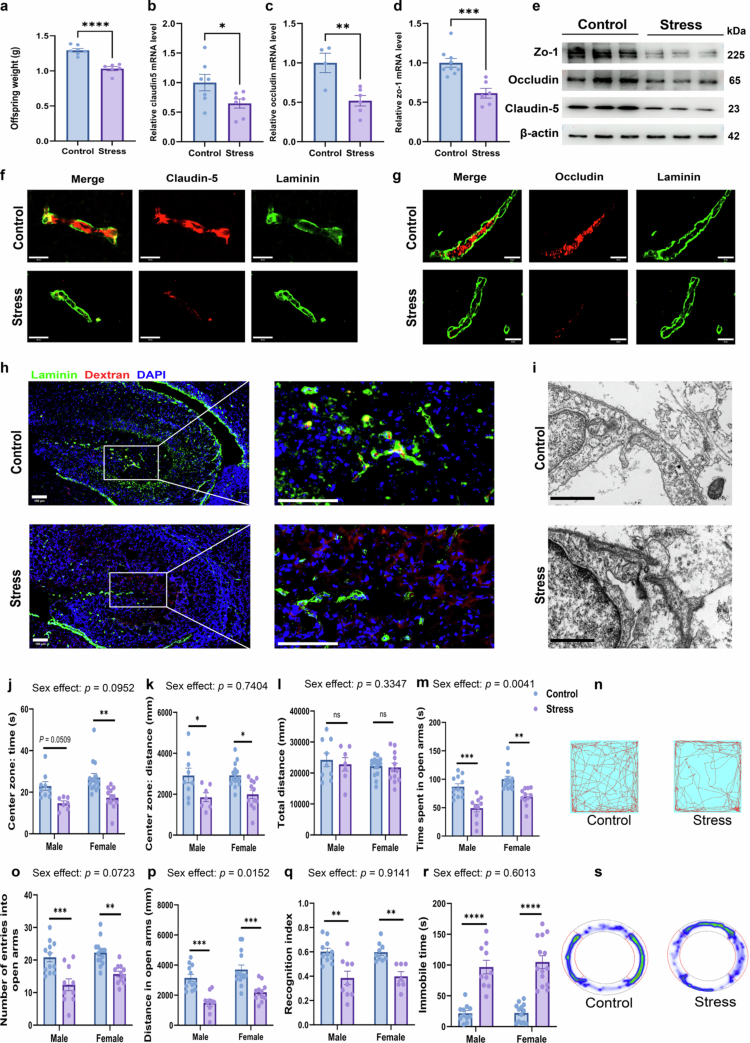
Prenatal stress induces fetal BBB developmental defects and abnormal behaviors in adult offspring of both sexes. (a) Offspring exposed to prenatal stress (*n* = 6) exhibited a significantly lower mean birth weight (****p < 0.0001) compared to the control group (*n* = 7). (b) qPCR analysis revealed significant downregulation of the *Claudin-5* gene in the fetal brains of prenatal stress offspring compared to controls (*n* = 7 per group and *p = 0.0463). (c) qPCR analysis revealed significant downregulation of the *Occludin* gene in the fetal brains of the prenatal stress offspring compared to controls (*n* = 4 control, 6 stress, and **p = 0.0058). (d) qPCR analysis revealed significant downregulation of the *ZO-1* gene in the fetal brains of the prenatal stress offspring Compared to controls (*n* = 9 control, 6 stress, and ***p = 0.0006). (e) Representative Western blot images demonstrate protein expression levels of key BBB components-Claudin-5, Occludin, and ZO-1, in fetal brains from both offspring groups. (f) Representative immunofluorescence images of the blood–brain barrier key protein Claudin-5 in fetal brains of both offspring groups, with endothelial cells labeled by green fluorescence (scale bar = 20 μm). (g) Representative immunofluorescence images of the blood–brain barrier key protein Occludin in fetal brains of both offspring groups (scale bar = 20 μm). (h) Representative images of blood‒brain barrier permeability assessment in the hippocampus of both groups, with 10 kDa dextran visualized by red fluorescence. The stress offspring exhibited diffuse red fluorescence signals, indicating compromised blood‒brain barrier integrity (scale bar = 100 μm). (i) Representative transmission electron microscopy images of blood‒brain barrier ultrastructure in fetal brains from both offspring groups. Stress offspring exhibited blurred tight junctions in fetal brain endothelial cells (scale bar = 10 μm). (j) Prenatal stress offspring of both sexes spent less time in the center zone of the open field, reflecting increased anxiety-like behavior (*n* = 9 male/control, 7 male/stress, 15 female/control, 12 female/stress, *p* = 0.0509 for male, and **p = 0.0012 for female). (k) Prenatal stress offspring of both sexes traveled shorter distance in the center zone of the open field (*n* = 9 male/control, 7 male/stress, 15 female/control, 12 female/stress, *p = 0.0318 for male, *p = 0.0124 for female). (l) No significant difference was observed in the total distance traveled between the prenatal stress group and the control group (*p* = 0.9339 for male, *p* = 0.9948 for female). (m, o, and p) In the elevated zero maze, offspring of both sexes from the stress group showed significantly reduced time spent in the open arms (m, ***p = 0.0002 for male, and **p = 0.0012 for female), fewer entries into the open arms (o, ***p = 0.0003 for male, and **p = 0.0034 for female), and decreased distance (p, ***p = 0.0002 for male, and ***p = 0.0004 for female) traveled compared to controls, indicating elevated anxiety levels (*n* = 12 male/control, 10 male/stress, 14 female/control, and 11 female/stress). (n) Representative open field test trajectories showed denser path distribution in the central zone for control offspring compared to stress groups. (q) Prenatal stress offspring of both sexes exhibited significantly lower cognitive indices, indicating impaired cognitive function (*n* = 11 male/control, 9 male/stress, 9 female/control, 7 female/stress, **p = 0.0010 for male, and **p = 0.0081 for female). (r) Prenatal stress offspring of both sexes demonstrated significantly increased immobility time in the tail suspension test, which is indicative of a depression-like phenotype (*n* = 11 male/control, 10 male/stress, 13 female/control, 14 female/stress, and ****p < 0.0001 for both sex). (s) Representative heat map trajectories of offspring from both groups in the elevated zero maze experiment. The open arms were highlighted in red. The data were presented as mean ± SEM. For the behavioral assays, each experimental group consisted of offspring derived from at least 6–8 distinct dams. *p < 0.05, **p < 0.01, ***p < 0.001, and ****p < 0.0001; ns, no significant difference. Statistical differences were determined by unpaired two-tailed *t*-tests (a–d) and two-way ANOVA with Sidak's multiple-comparison test (j–m, o–r).

Prenatal stress significantly reduced offspring birth weight (*p* < 0.0001, Figure 2a), indicating impaired fetal development. To assess BBB structural integrity, we examined the expression of tight junction components in E18.5 fetal brains. qPCR analysis revealed significantly downregulated mRNA expression of Cldn5 (Claudin-5), Ocln (Occludin), and Tjp1 (ZO-1) in stress-exposed fetuses compared to controls (all *p* < 0.05, Figure 2b–d). These findings were confirmed at the protein level, where Western blotting showed consistent reductions in these markers (Figure 2e), and immunofluorescence demonstrated diminished Claudin-5 and Occludin immunoreactivity (Figure 2f and g).

Functionally, hepatic tracer injection of 10-kDa dextran‒tetramethylrhodamine revealed extensive BBB leakage in the stress group. While the tracer was strictly confined to the vasculature in control fetuses, stress-exposed offspring exhibited significant parenchymal extravasation in the hippocampus (Figure 2h), hypothalamus (Supplementary Figure S1a), and prefrontal cortex (Supplementary Figure S1b). Corroborating these functional deficits, transmission electron microscopy showed diffuse and disorganized tight junction ultrastructure in the stress group, contrasting with the well-defined junctions in controls (Figure 2i).

We next evaluated long-term behavioral consequences in 6-week-old adult offspring. In the OFT, offspring of both sexes from the stress group spent less time (Figure 2j) and traveled shorter distances (*p* < 0.05; Figure 2k) in the center zone, with no significant difference in total locomotion (Figure 2l). Regarding time spent and distance traveled in the central area of the open field, no effect of sex (*F*_1,39_ = 2.924, *p* = 0.0952 and *F*_1,39_ = 0.1114, *p* = 0.7404, respectively) or group × sex interaction (*F*_1,39_ = 0.1314, *p* = 0.7189 and *F*_1,39_ = 0.0908, *p* = 0.7647, respectively) were observed. Anxiety-like phenotypes were further confirmed in the EZM, where stress offspring exhibited significant reductions in time spent (*p* < 0.01, Figure 2m), number of entries (*p* < 0.01, Figure 2o), and distance traveled (*p* < 0.001, Figure 2p) in the open arms compared to the control group. Regarding time spent and distance traveled in the open arms, a main effect of sex was found (*F*_1,43_ = 9.186, *p*= 0.0041 and *F*_1,43_ = 6.397, *p* = 0.0152, respectively). Thus, stress affected anxiety-like behavior, and more importantly, both sexes were differently affected. Anxiety level was higher in males than in females within the stress group. Additionally, stress-exposed offspring displayed cognitive deficits in the NOR test, as indicated by a significantly lower preference for the novel object (*p* < 0.01, Figure 2q). Depressive-like behavior was also evident, as the stress group showed prolonged immobility time in the TST (*p* < 0.0001, Figure 2r).

### Maternal probiotic supplementation ameliorates stress-induced gut microbiota dysbiosis and fetal BBB developmental impairments

Given that stress-exposed dams exhibited gut dysbiosis concurrent with offspring BBB developmental impairments, we investigated whether probiotic-mediated microbiota modulation could rescue these defects. We found that maternal probiotic supplementation effectively alleviated stress-induced anxiety-like behaviors in dams. This was evidenced by the significantly increased distance traveled (*p* = 0.0443, Supplementary Figure S2a) and time spent (*p* = 0.0434, Supplementary Figure S2b) in the central zone of the open field compared to the stress group. Importantly, total locomotion remained comparable across all three groups (*F*_2,17_ = 0.3577, *p* = 0.7044, Supplementary Figure S2c), ruling out locomotor deficits. No significant differences were observed in gestational weight gain among the three groups (*F*_2,17_ = 0.2326, *p* = 0.7950, Supplementary Figure S2d). Histological examination via H&E staining demonstrated that probiotic treatment prevented stress-induced intestinal injury, maintaining an intact villus architecture comparable to that of the controls (Supplementary Figure S2e).

**Figure 3.**
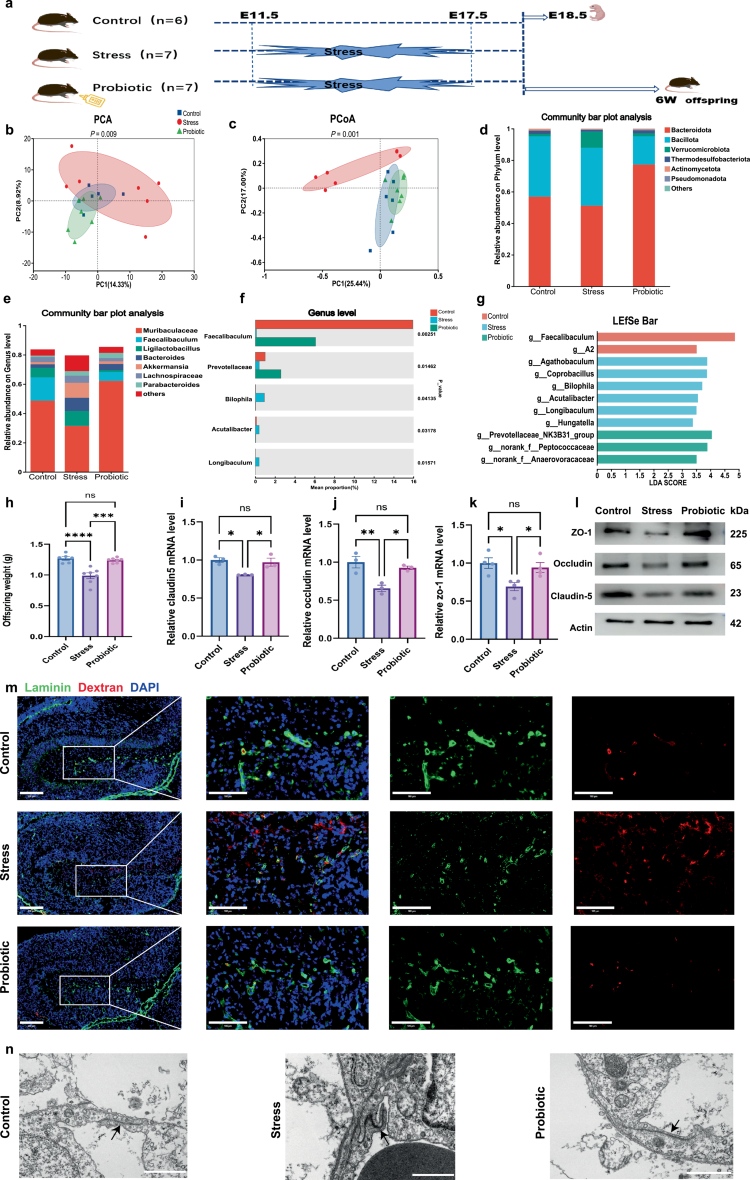
Maternal probiotic supplementation restores gut microbiota dysbiosis and reverts BBB developmental defects in stress-exposed offspring. (a) Schematic timeline of the experimental procedure. The experiment included three groups, with the addition of a prenatal probiotic supplementation group, while maintaining the same maternal stress protocol as before. (b and c) PCA (b, **p = 0.009) and PCoA (c, ***p = 0.001) of fecal gut microbiota sequencing from three groups of mice (*n* = 6 control, 7 stress, and 7 probiotic) at the ASV level. (d and e) Community bar plot analysis of the gut microbiota sequencing of fecal samples from three groups of mice (*n* = 6 control, 7 stress, and 7 probiotic) at the phylum level (d) and genus level (e). (f) Kruskal–Wallis H test bar plot on genus level from three groups of mice (*n* = 6 control, 7 stress, and 7 probiotic). (g) LEfSe analysis of differentially abundant microbial taxa among the three groups of mice (*n* = 6 control, 7 stress, and 7 probiotic). (h) Maternal probiotic supplementation significantly increased the birth weight of offspring exposed to prenatal stress (****p < 0.0001 control vs stress and ***p = 0.0001 stress vs probiotic). (i–k) qPCR analysis revealed that the probiotic supplementation group exhibited upregulated expression of key BBB tight junction proteins—*Claudin-5* (i, *p = 0.0378), *Occludin* (j, *p = 0.0254), and *ZO-1* (k, *p = 0.0477). (l) Western blot analysis of key blood–brain barrier tight junction proteins across the three groups. (m) Representative immunofluorescence images of fetal BBB permeability across the three groups: red signals indicate tracers, while green labels mark endothelium. Probiotic supplementation ameliorated stress-induced BBB leakage (scale bar = 200 μm or 100 μm). (n) Representative electron microscopy images of tight junction structures in the fetal blood‒brain barrier across the three groups (scale bar = 10 μm). The data were presented as mean ± SEM. *p < 0.05, **p < 0.01, ***p < 0.001, and ****p < 0.0001. Statistical differences were determined by one-way ANOVA with Tukey's multiple-comparison test (h–k).

Regarding the gut microbiome, alpha-diversity indices showed no significant differences among the three groups (Supplementary Figure S2f–h). However, beta-diversity analysis revealed distinct community structures. Principal coordinate analysis (PCoA) based on Bray‒Curtis distances demonstrated that the probiotic group clustered closely with controls, segregating from the stress-exposed group (*p* = 0.001, Figure 3c). This pattern suggested that probiotic intervention may promote partial restoration of gut microbial communities toward a normative state. At the phylum level, probiotic supplementation increased the relative abundance of Bacteroidetes while decreasing Bacillota (formerly Firmicutes) compared to the stress group (Figure 3d). At the genus level, *Muribaculaceae* and *Fecalibaculum* were enriched following probiotic treatment (Figure 3e). The Kruskal‒Wallis H test also confirmed statistically significant differences in *Fecalibaculum* abundance across the three groups (*p* = 0.0025, Figure 3f). LEfSe analysis further identified specific taxonomic biomarkers: the stress group was enriched in *Bilophila* (LDA > 3), whereas the probiotic group was characterized by an enrichment of *Prevotellaceae* (Figure 3g), which could mitigate intestinal barrier injury and microbiota dysbiosis.^22^ These data demonstrate that maternal probiotic supplementation ameliorates stress-induced gut microbiota dysbiosis.

Importantly, maternal probiotic intervention exerted protective effects on offspring development. Probiotic supplementation normalized offspring birth weight, which was significantly reduced by prenatal stress (*p* = 0.0001, Figure 3h). In E18.5 fetal brains, qPCR analysis showed that probiotic treatment rescued the expression of tight junction genes, including Cldn5, Ocln, and Tjp1, restoring levels to those observed in controls (all *p* < 0.05, Figure 3i–k). To further characterize BBB development, we assessed BBB permeability in offspring from all three groups. While the stress group displayed significant extravascular leakage of the 10-kDa dextran tracer in the hippocampus, the probiotic group exhibited intact vascular confinement, indicating preserved BBB integrity (Figure 3m). Consistent with these functional findings, transmission electron microscopy confirmed that probiotic-exposed offspring maintained a well-defined endothelial tight junction ultrastructure (Figure 3n). These findings illustrate that stress-induced offspring BBB developmental defects are reversible, with the maternal gut microbiota serving as a key potential regulatory factor.

### Maternal probiotic supplementation alleviates stress-induced fetal neuroinflammation and microglial activation

**Figure 4.**
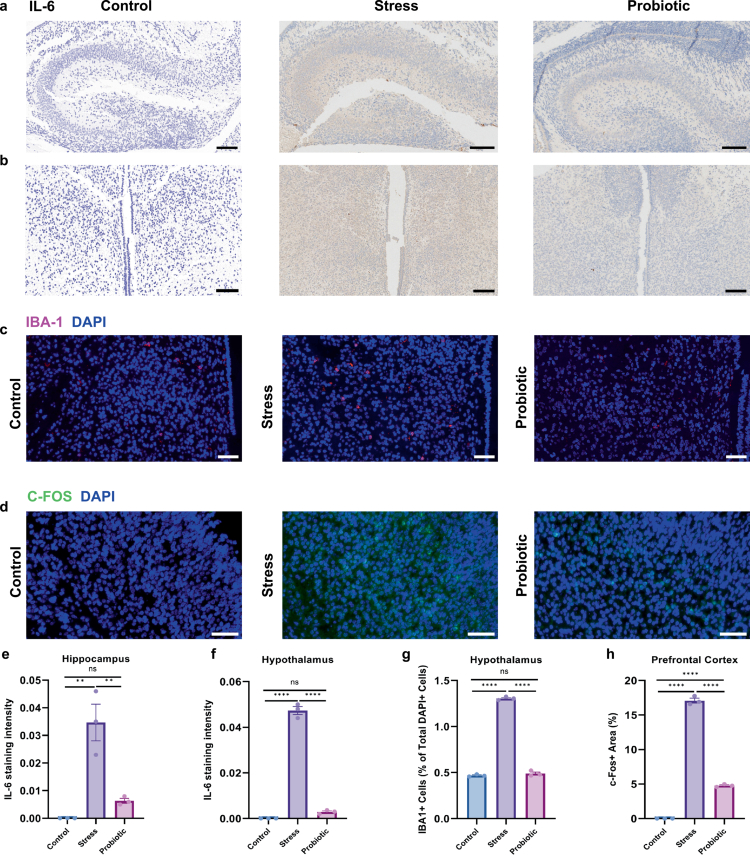
Maternal probiotic supplementation ameliorates stress-induced fetal neuroinflammation and microglial activation. (a) Representative IHC images of IL-6 in the fetal hippocampus. Scale bar = 100 μm. (b) Representative IHC images of IL-6 in the fetal hypothalamus. Scale bar = 100 μm. (c) Representative images of IBA1 (red) and DAPI (blue) in the fetal hypothalamus. Scale bar = 50 μm. (d) Representative images of c-Fos immunostaining (green) in the fetal prefrontal cortex. Scale bar = 50 μm. (e and f) Quantification of IL-6 expression by mean optical density (MOD). MOD quantification revealed elevated IL-6 expression in the hippocampal (**p = 0.0016) and hypothalamic (****p < 0.0001) regions of prenatally stressed offspring, which was reduced following probiotic supplementation. (g) Quantification showing the percentage of IBA1+ microglia relative to total DAPI+ nuclei (****p < 0.0001). (h) Quantification of c-Fos+ area percentage (****p < 0.0001). The data were presented as mean ± SEM. *p < 0.05, **p < 0.01, ***p < 0.001, and ****p< 0.0001; statistical differences were determined by one-way ANOVA with Tukey's multiple-comparison test (e–h).

BBB dysfunction amplifies neuroinflammation.^23^ To determine if probiotic treatment mitigates stress-induced neuroinflammation, we quantified cytokine expression in E18.5 fetal brains. Immunohistochemical analysis revealed that prenatal stress significantly upregulated IL-6 expression in the hippocampus (*p* = 0.0018, Figure 4a) and hypothalamus (*p* < 0.0001, Figure 4b) compared to controls. Importantly, maternal probiotic supplementation effectively reversed this effect. Quantitative analysis showed no significant difference in IL-6 expression between the probiotic and control groups in the hippocampus (*p* = 0.5199, Figure 4e) and hypothalamus (*p* = 0.2170, Figure 4f). Similarly, the IL-1β levels were significantly elevated in the stress group in both hippocampal (*p* < 0.0001, Supplementary Figure S3a) and hypothalamic regions (*p* = 0.0002, Supplementary Figure S3b) compared to controls. While probiotic supplementation significantly reduced IL-1β expression compared to the stress group (*p* = 0.008 and *p* = 0.0318, respectively), the levels remained partially elevated Compared to controls (Supplementary Figure S3c and d).

Microglia are known to be key drivers of neuroinflammation.^24^ We next examined microglial status in the fetal hypothalamus. The stress group exhibited aberrant microglial proliferation and clustering compared to controls (*p* < 0.0001, Figure 4c). Notably, probiotic intervention normalized microglial numbers, with quantitative density analysis revealing no significant differences between the probiotic and control groups (*p* = 0.4545, Figure 4g). Furthermore, we assessed neuronal activation in the fetal prefrontal cortex (PFC) using c-Fos. While basal c-Fos expression was low in the controls, prenatal stress induced a significant upregulation (*p* < 0.0001). This stress-induced cortical activation was significantly attenuated by maternal probiotic supplementation (*p* < 0.0001, Figure 4d). Collectively, these findings demonstrate that maternal probiotic supplementation suppresses fetal neuroinflammation and normalizes aberrant microglial and neuronal activation.

### Maternal probiotic supplementation reverses stress-induced behavioral deficits and BBB dysfunction in adult offspring

To determine if stress-induced behavioral deficits in adult offspring were rescued by maternal probiotic supplementation, we assessed a series of behavioral tests in adult offspring of both sexes.

**Figure 5.**
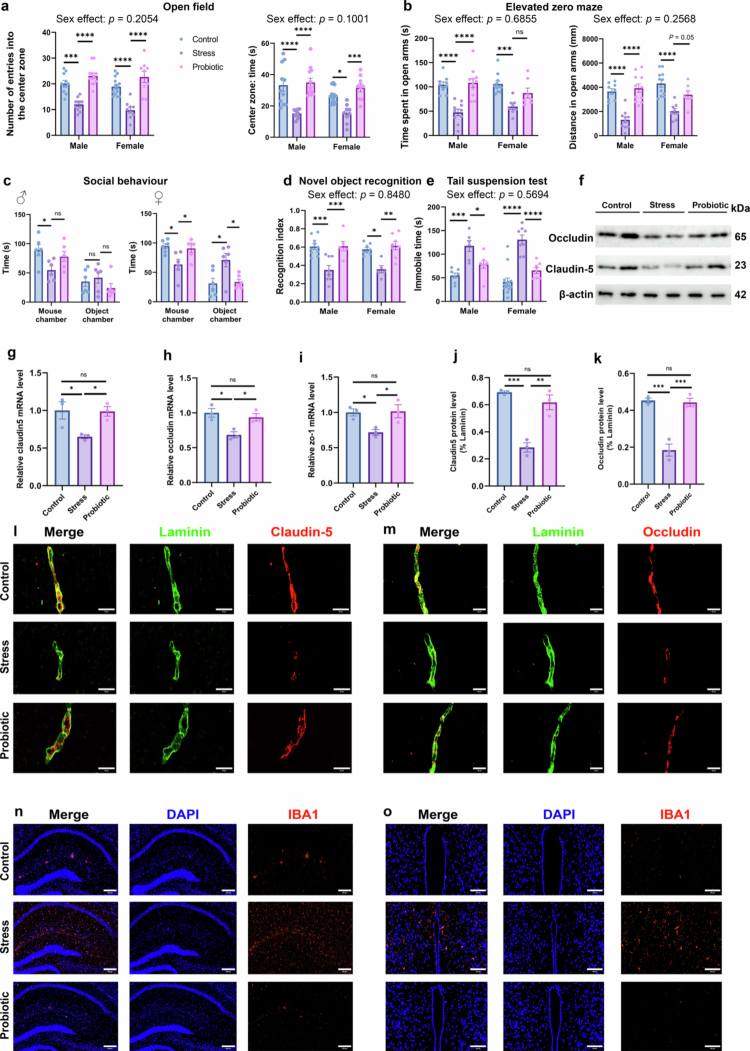
Maternal probiotic supplementation reverses stress-induced behavioral deficits and BBB dysfunction in adult offspring. (a) Frequency and duration of central zone entries in the open field test of adult offspring from three groups. Offspring from the stress group exhibited significantly reduced entries (***p = 0.0002 for male and ****p < 0.0001 for female) and time spent (****p < 0.0001 for male and *p = 0.0171 for female) in the central zone compared to controls, but these parameters were restored in the probiotic group (*n* = 11 male/control, 11 male/stress, 12 male/probiotic, 13 female/control, 9 female/stress, and 9 female/probiotic). (b) Duration and distance of open arm entries in the elevated zero maze of adult offspring from the three groups. Offspring in the probiotic group exhibited a significant increase in the time spent in the open arms (****p < 0.0001 for male) and distance traveled (****p < 0.0001 for male, *p* = 0.0533 for female) compared to the stress group, indicating that probiotic intervention ameliorates anxiety-like behaviors (*n* = 9 male/control, 10 male/stress, 11 male/probiotic, 11 female/control, 8 female/stress, and 7 female/probiotic). (c) Social behavior and preference for social novelty were tested in the three-chambered apparatus (*n* = 6). (d) Cognitive indices in the novel object recognition test differed significantly among the three groups. Probiotic group demonstrated a significantly higher cognitive index compared to the stress group (***p = 0.001 for male and **p = 0.0023 for female), indicating enhanced cognitive performance (*n* = 10 male/control, 8 male/stress, 6 male/probiotic, 8 female/control, 5 female/stress, and 8 female/probiotic). (e) Immobility time in the tail suspension test differed significantly among the three groups. Probiotic supplementation substantially reduced immobility duration compared to the stress group (*p = 0.0488 for male and ****p < 0.0001 for female), demonstrating improved depression-like phenotypes (*n* = 7 male/control, 6 male/stress, 8 male/probiotic, 14 female/control, 8 female/stress, and 7 female/probiotic). (f) Western blot analysis of key BBB tight junction proteins across the three groups. (g–i) qPCR analysis revealed that adult offspring from the probiotic group exhibited upregulated expression of key BBB tight junction proteins (*Claudin-5*, *Occludin*, and *ZO-1*) compared to the stress group (*n* = 3 per group). (j and k) Quantitative analysis of Claudin-5 (j) and Occludin (k) immunofluorescence across the three groups (*n* = 3 per group). (l and m) Representative immunofluorescence images of Claudin-5 (red) and Occludin (red) in adult offspring across the three groups. Scale bar = 20 μm. (n) Representative images of IBA1 (red) and DAPI (blue) in adult offspring hippocampus. Scale bar = 200 μm. (o) Representative images of IBA1 (red) and DAPI (blue) in adult offspring hypothalamus. Scale bar = 100 μm. The data were presented as mean ± SEM. For the behavioral assays, each experimental group consisted of offspring derived from at least 6–8 distinct dams. *p < 0.05, **p < 0.01, ***p < 0.001, and ****p < 0.0001; ns, no significant difference. Statistical differences were determined by two-way ANOVA with Sidak's multiple-comparison test (a–e) and one-way ANOVA with Tukey's multiple comparison test (g–k).

Importantly, we analyzed the data using two-way ANOVA to evaluate potential sex differences. In the open field test (OFT), results showed no main effect of sex (*F*_*1,59*_ = 1.639, *p* = 0.2054 and *F*_1,59_ = 2.791, *p* = 0.1001, respectively) or group × sex interaction (*F*_*2,59*_ = 0.2646, *p* = 0.7684 and *F*_2,59_ = 0.9578, *p* = 0.3896, respectively) on central zone parameters. However, a significant main effect of group was observed (*F*_*2,59*_ = 49.28, *p* < 0.0001 and *F*_2,59_ = 30.09, *p* < 0.0001, respectively). Post-hoc analysis revealed that maternal probiotic supplementation significantly ameliorated anxiety-like behaviors; the probiotic group exhibited increased frequency (all *p* < 0.0001, Figure 5a) and duration (*p* < 0.0001 for male and *P* = 0.0007 for female, Figure 5a) in the central area compared to the stress group. In the elevated zero maze (EZM), for time spent and distance traveled in the open arms, no significant main effects of sex (*F*_1,50_ = 0.1659, *p* = 0.6855 and *F*_1,50_ = 1.316, *p* = 0.2568, respectively) or interactions (*F*_2,50_ = 2.402, *p* = 0.1009 and *F*_2,50_ = 2.782, *p* = 0.0715, respectively) were found. Compared to the stress group, the probiotic group exhibited significantly increased distances in males (*p* < 0.0001) and a trend toward increased distances in females (*p* = 0.0533, Figure 5b); no significant differences were observed between the probiotic and control groups. The mice were also tested in the three-chamber apparatus for social interactions. Sociability is defined as the experimental mouse spending more time in the chamber containing the novel mouse than in that containing the novel object. Analysis revealed a main effect of group (*F*_2,30_ = 10.04, *p* = 0.0005) on the time spent in the novel mouse chamber but no main effect of sex (*F*_1,30_ = 2.109, *p* = 0.1568) and no significant group × sex interaction (*F*_2,30_ = 0.1140, *p* = 0.8927). Post hoc tests showed that stressed mice spent less time in the novel mouse chamber compared to controls (*p* = 0.0294 for male and *p* = 0.0141 for female, Figure 5c, Supplementary Figure S4c and d). These results show that prenatal stress reduced social behavior of adult offspring. However, maternal prenatal intake of probiotics prevented this reduction of sociability in females (*p* = 0.0308, Figure 5c). Mice were then tested for preference for social novelty by assessing the time spent with a novel stranger or the initial stranger, strangers 1 and 2, respectively (Supplementary Figure S4a and b). Two-way ANOVA revealed a main effect of group (*F*_2,30_ = 8.508, *p* = 0.0012) but not sex (*F*_1,30_ = 2.095, *p* = 0.1582) or group × sex interaction (*F*_2,30_ = 0.0890, *p* = 0.9150) on the time spent in the chambers containing stranger 2. When analysed separately for males and females, the preventive effect of probiotic supplementation was more pronounced in males (Supplementary Figure S4b). Regarding cognitive function, the novel object recognition test indicated a significant group effect (*F*_*2,39*_ = 22.86, *p* < 0.0001) that was independent of sex (*F*_*1,39*_ = 0.0372, *p* = 0.8480). The discrimination index in stressed offspring was significantly restored following probiotic supplementation (*p* = 0.001 for male and *p* = 0.0023 for female, Figure 5d). Furthermore, in the tail suspension test, the probiotic group exhibited significantly reduced immobility time compared to the stress group (*p* = 0.0488 for male and *p* < 0.0001 for female, Figure 5e), suggesting an alleviation of depression-like behavior in both sexes.

Prenatal stress resulted in persistent BBB deficits in adult offspring. Western blot and qPCR analyses revealed significantly downregulated expression of critical tight junction components (Claudin-5, Occludin, and ZO-1) of the stress group compared to controls (*p* < 0.05; Figure 5f–i). Notably, maternal probiotic supplementation effectively rescued this impairment, restoring the expression of these markers to levels comparable to those of the controls. Consistent with these molecular findings, immunofluorescence imaging demonstrated that while stress-exposed adults exhibited diminished vascular Claudin-5 and Occludin immunoreactivity, probiotic-treated offspring maintained an intact junctional architecture (Figure 5j–m). Paralleling the BBB outcome, adult offspring from the stress group exhibited significantly increased microglial density in both the hippocampus (Figure 5n) and hypothalamus (Figure 5o). Collectively, these data demonstrate that maternal probiotic supplementation exerts long-lasting protective effects, mitigating both stress-induced BBB dysfunction and microglial hyperactivation in adulthood.

### Prenatal stress suppresses IFN-β signaling in the placental–fetal brain axis

**Figure 6.**
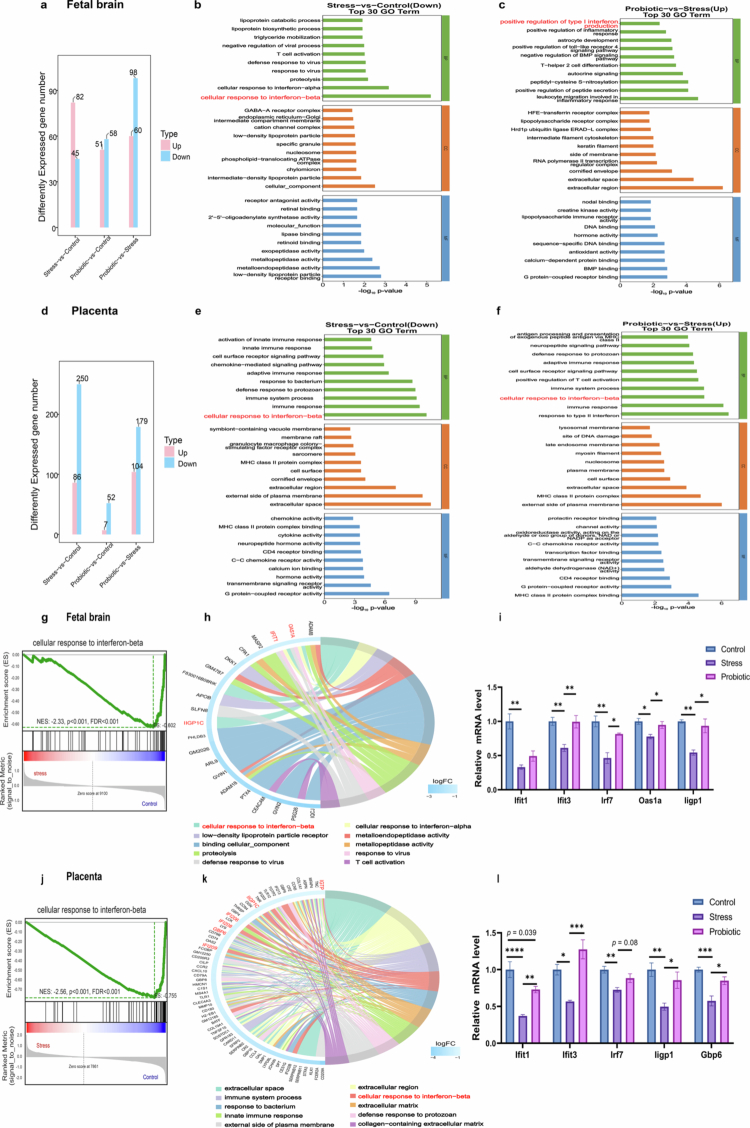
Prenatal stress down-regulates IFN-β signaling in the placental–fetal brain axis. (a) Bar graph illustrating the statistical distribution of significantly upregulated and downregulated genes in fetal brains across three offspring groups (*n* = 8 control, 6 stress, and 6 probiotic). (b) Gene ontology enrichment analysis of differentially expressed genes in fetal brain tissues between the stress and control offspring groups revealed significantly downregulated pathways, including cellular response to interferon-β (*n* = 8 control, 6 stress, and 6 probiotic). (c) Gene ontology enrichment analysis of differentially expressed genes in offspring fetal brain tissues between the probiotic group and stress group identified significantly upregulated pathways, including positive regulation of the inflammatory response and type I interferon production (*n* = 8 control, 6 stress, and 6 probiotic). (d) Bar graph illustrating the statistical distribution of significantly upregulated and downregulated genes in the placenta across the three offspring groups (*n* = 8 control, 6 stress, and 6 probiotic). (e) Gene Ontology enrichment analysis of differentially expressed genes in placental tissues between the stress and control groups revealed significantly downregulated pathways, including cellular response to interferon-β (*n* = 8 control, 6 stress, and 6 probiotic). (f) Gene Ontology enrichment analysis of differentially expressed genes in placental tissues between the probiotic group and stress group revealed significantly upregulated pathways, including cellular response to interferon-β (*n* = 8 control, 6 stress, and 6 probiotic). (g) GSEA (gene set enrichment analysis) confirmed the downregulation of the IFN-β signaling pathway in the fetal brain of the stress group compared to controls. (h) The chord diagram shows the differential genes of the cellular response to IFN-β pathway down-regulated in the fetal brain of the stress group, including *Oas1a*, *Ifit1*, and *Iigp1c*. (i) qPCR analysis revealed downregulation of a series of IFN-β related signaling genes in the fetal brain of prenatally stressed offspring (*n* = 3–5 per group), while probiotic supplementation restored its expression. (j) GSEA confirmed the downregulation of the IFN-β signaling pathway in the placenta of the stress group compared to controls. (k) The chord diagram shows the differential genes of the cellular response to IFN-β pathway down-regulated in placenta of the stress group, including *Gbp6*, *Iigp1c*. (l) qPCR analysis revealed the downregulation of a series of IFN-β related signaling genes in the placenta of prenatally stressed offspring (*n* = 3‒5 per group), while maternal probiotic supplementation upregulated their expression. The data were presented as mean ± SEM. The sample size (n) reflects the number of litters. *p < 0.05, **p < 0.01, ***p < 0.001, and ****p < 0.0001. Statistical differences were determined by one-way ANOVA with Tukey's multiple-comparison test (i and l).

Transcriptomic profiling of E18.5 placental and fetal brain tissues revealed widespread gene expression changes induced by maternal stress and probiotic intervention. In the fetal brain, we identified 127 differentially expressed genes (DEGs) between the stress and control groups and 158 DEGs between the probiotic and stress groups (Figure 6a). Parallel analysis in the placenta identified 336 and 283 DEGs, respectively (Figure 6d).

Functional analysis revealed a synchronized disruption of interferon signaling across the axis. In both fetal brain and placental tissues, Gene Ontology (GO) enrichment analysis indicated that pathways associated with the cellular response to interferon-β were significantly downregulated in the stress group (Figure 6b and e). Conversely, the probiotic group showed significant upregulation of pathways related to the positive regulation of type I interferon production or the cellular response to interferon-β relative to the stress group (Figure 6c and f). These findings were further corroborated by gene set enrichment analysis (GSEA), which confirmed the suppression of interferon-β signaling in the stress group in both fetal brain (*p* < 0.001, Figure 6g) and placenta (*p* < 0.001, Figure 6j). Chord diagrams highlighted specific downregulated DEGs within this pathway, including *Ifit1*, *Oas1a*, and *Iigp1c* in the fetal brain (Figure 6h) and *Iigp1c*, *Ifi206*, *Ifi208*, *Ifi209*, and *Gbp6* in the placenta (Figure 6k).

To validate the transcriptomic findings, we quantified the expression of key IFN-β pathway components via qPCR. Consistent with the RNA-seq data, the stress group exhibited significantly reduced expression of *Ifit1*, Ifit3, *Irf7*, *Oas1a*, and *Iigp1* in the fetal brain (Figure 6i) compared to controls. Importantly, maternal probiotic supplementation effectively restored the expression of these genes. Similarly, in the placenta, stress significantly downregulated *Ifit1*, *Ifit3*, *Irf7*, *Iigp1*, and *Gbp6*, whereas probiotic treatment successfully reversed these deficits (Figure 6l). To assess the regulatory role of the IFN-β signaling pathway on tight junction proteins (TJPs), endothelial cells were transfected with siRNA targeting *Ifnar1* (si-*Ifnar1*) or a negative control (si-NC). Transfection with si-*Ifnar1* significantly suppressed *Ifnar1* expression compared to the si-NC group (*p* < 0.0001, Supplementary Figure S5a), confirming efficient knockdown. Importantly, *Ifnar1* silencing led to the downregulation of Claudin-5 and ZO-1 expression compared to the si-NC group (Supplementary Figure S5b and d).

### Prenatal stress alters maternal tryptophan metabolism

**Figure 7.**
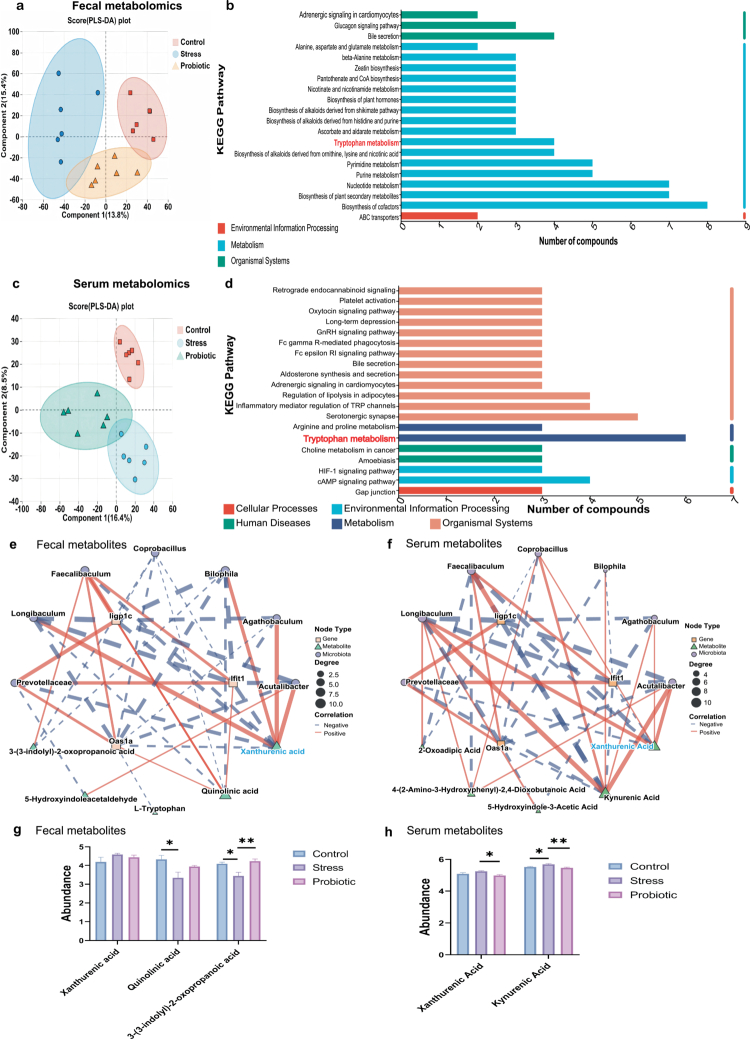
Prenatal stress exposure profoundly influences the maternal fecal and serum tryptophan metabolism. (a) PLS‒DA (partial least squares discriminant analysis) of maternal fecal metabolomics across the three experimental groups revealed distinct metabolic profiles (*n* = 6 per group, **p = 0.007). (b) KEGG pathway analysis of differential metabolites in three groups of fecal samples. (c) PLS–DA of maternal serum metabolomics across the three experimental groups revealed distinct metabolic profiles (*n* = 6 per group, **p = 0.003). (d) KEGG pathway analysis of differential metabolites in three groups of maternal serum samples. (e) Interaction network between differentially abundant maternal gut species, differentially abundant fecal metabolites, and mRNA expression related to fetal brain development. Triangular nodes represent metabolites, while circular nodes represent bacterial species, and square nodes represent the fetal brain differential genes. The size of the nodes indicates the degree of the network. Red edges represent positive correlations; blue edges represent negative correlations. (f) Similar to (e), an interaction network was created between differentially abundant maternal species, differentially abundant serum metabolites, and mRNA expression related to fetal brain development. (g and h) Post-hoc analysis of metabolites related to tryptophan metabolism in fecal samples (g, *p = 0.0128 for quinolinic acid, *p = 0.0138 and **p = 0.0032 for 3-(3-Indolyl)-2-oxopropanoic acid) and maternal serum samples (h, *p = 0.0142 for xanthurenic acid, *p = 0.0421 and **p = 0.0073 for kynurenic acid). *n* = 6 mice/group. The data were presented as mean ± SEM. *p < 0.05 and **p < 0.01. Correlation analysis was performed using the Spearman's rank correlation test. Statistical differences were determined by one-way ANOVA with Tukey's multiple-comparison test (g and h).

To elucidate the metabolic mechanisms linking the maternal gut microbiota to fetal brain development, we performed liquid chromatography‒mass spectrometry (LC‒MS)-based untargeted metabolomics on maternal fecal and serum samples. Multivariate statistical analysis revealed distinct metabolic profiles across the three groups in both fecal (PERMANOVA, *p* = 0.007, Figure 7a) and serum samples (PERMANOVA, *p* = 0.003, Figure 7c). Notably, KEGG pathway enrichment analysis identified tryptophan metabolism as a shared pathway significantly altered by prenatal stress in both matrices (Figure 7b and d).

To identify key metabolic mediators, we constructed a multi-omics correlation network integrating differentially abundant maternal bacteria, tryptophan metabolites, and the expression of IFN-β signaling genes (*Iigp1*, *Oas1a*, *Ifit1*) in the fetal brain. We observed significant positive correlations between the beneficial taxa (*Fecalibaculum* and *Prevotellaceae*) and the expression of these genes (Figure 7e). Conversely, specific tryptophan catabolites showed negative associations. Detailed quantification revealed tissue-specific dynamics. In the feces, while the abundance of xanthurenic acid was highest in the stress group, the difference did not reach statistical significance (*p* > 0.05, Figure 7g). However, in maternal serum, the levels of kynurenic acid and xanthurenic acid were significantly elevated in the stress group (Figure 7h). Importantly, correlation network analysis highlighted that these serum metabolites were negatively associated with both beneficial gut bacteria and fetal brain IFN-β gene expression (Figure 7f). Furthermore, maternal probiotic supplementation effectively reversed the stress-induced elevation of serum kynurenic acid and xanthurenic acid levels (Figure 7h). These data indicate that prenatal stress disrupts maternal tryptophan metabolism, leading to an accumulation of circulating kynurenic and xanthurenic acids, which correlates with suppressed fetal neuroimmune signaling. To further investigate the functional role of this metabolic pathway, we treated bEnd.3 cells with 1-MT (an IDO/TDO inhibitor) following *Ifnar1* knockdown. Notably, 1-MT treatment effectively attenuated the downregulation of tight junction proteins (*Claudin-5* and *ZO-1*) induced by *Ifnar1* silencing (Supplementary Figure S5b and d). This suggests that inhibiting tryptophan catabolism exerts a protective effect on endothelial integrity under conditions of compromised IFN-β signaling.

## Discussion

In summary, our findings demonstrate that prenatal stress not only induces behavioral abnormalities in adult offspring but also critically impairs fetal BBB development, identifying the maternal gut microbiota as a pivotal mediator of these deficits. Crucially, we show that these neurodevelopmental defects are reversible; maternal probiotic supplementation effectively rescues stress-induced BBB dysfunction, microglial hyperactivation, and subsequent behavioral phenotypes. Mechanistically, this rescue effect is underpinned by the restoration of the IFN-β signaling pathway. We propose that under stress conditions, the accumulation of deleterious gut-derived tryptophan metabolites suppresses physiological IFN-β expression, thereby precipitating neurodevelopmental deficits. Collectively, these insights advance our understanding of the maternal gut–placenta–fetal brain axis and highlight the therapeutic potential of targeting the maternal microbiome to prevent psychiatric disorders associated with prenatal stress.

The BBB constitutes a critical component of the neurovascular unit, regulating substance entry and immune cell trafficking into the CNS parenchyma, thereby protecting neural homeostasis.^25^ BBB maturation occurs progressively during gestation, with integrity essential for normal CNS function. Studies using GF mice show that a more permeable BBB is observed in fetal mice with GF mothers at E16.5 to E18.5 days of embryonic development compared to the fetal mice with pathogen-free mothers at equivalent gestational stages.^10^ Furthermore, low-dose penicillin exposure in late pregnancy and early postnatal elevates proinflammatory cytokine expression in the adult offspring prefrontal cortex, compromises BBB integrity, and induces behavioral alterations.^26^ Our data align with these observations, demonstrating that prenatal stress-induced maternal gut dysbiosis impairs fetal BBB development. Notably, the maternal gut microbiota influences offspring BBB integrity independently of offspring sex,^10^ though sex-specific differences in BBB development warrant further investigation.

BBB disturbances commonly occur in neuroinflammation and neurodegeneration.^27^ We observed upregulated inflammatory cytokine levels across multiple brain regions in prenatally stressed offspring. Chronic exposure to inflammatory mediators can disrupt tight junctions and integrins, promoting endothelial senescence and detachment.^28^ Several studies have shown that impaired permeability of the BBB due to stress is mediated by the translocation of proinflammatory factors (e.g., IL-1β, IL-17α, IL-23, and TNF-α) from the peripheral circulation to the BBB.^25^ Menard et al. demonstrated that BBB hyperpermeability in a chronic social defeat stress mouse model, promoting peripheral IL-6 passage across the BBB and depression.^4^ Another study reported that acute social defeat in stress-sensitized mice increased circulating monocytes and macrophage infiltration into the brain, subsequently activating brain endothelial cells and microglia, which in turn resulted in elevated production of IL-1β, IL-6 and TNF-α in the mouse brain. Notably, anxiety-like behaviors in the experimental animals correlated with the rise in proinflammatory cytokines.^29^^,^^30^ Cheng et al. also found that the BBB permeability increased in the hippocampus immediately after learned helplessness induction and that the levels of TNF-α, IL-17α, and IL-23 elevated with prolonged learned helplessness in mice.^31^ Interestingly, the elevated cytokine levels were attenuated by the TNF-α inhibitor etanercept, which ultimately mitigating the impaired BBB. These studies strongly suggest that proinflammatory signaling serves as a pivotal mediator of BBB disruption under stress conditions.

Probiotic treatment can reverse inflammation-related increased anxiety-like behavior in mice models.^32^ Our study extends this by demonstrating transgenerational effects: maternal probiotic supplementation attenuated anxiety-, depression-like behaviors, and cognitive deficits in prenatally stressed offspring. These improvements coincided with restored BBB integrity and reduced neuroinflammation across brain regions, indicating that maternal stress-induced offspring inflammation and BBB dysfunction are reversible and microbiota-dependent. This aligns with findings that maternal probiotic administration during maternal-separation stress mitigate offspring emotional disturbances.^12^

Microglia, the primary resident immune cells, are increasingly recognized as an early indicators of neuroinflammation and are essential for normal BBB development.^33^ Activated microglia produce various inflammatory mediators and subsequently induce neuroinflammation and neuronal damage.^34^ Microglia colonize the developing brain starting at E8.5 in mice and undergo distinct maturation stages that are intricately associated with dynamic changes in the CNS microenvironment. External perturbations may disrupt this tightly regulated developmental program, potentially leading to long-term functional consequences.^35^ Microglia respond to perturbations by initiating immune reactions, including the release of proinflammatory cytokines such as IL-6, IL-1β, and TNF-α, and can also be activated by peripherally derived cytokines that reach the brain.^36^^,^^37^ Here, we found that maternal stress not only activated microglia in the fetal hypothalamus but also induced sustained microglial hyperactivation in both the hippocampus and hypothalamus of adult offspring, concomitant with BBB dysfunction. Studies using minocycline injection in adult mice to suppress reactive microglial activation have demonstrated attenuation of LPS-induced BBB leakage, indicating that inhibiting microglial activation helps maintain BBB integrity.^38^ Prominently, our study found that maternal probiotic supplementation attenuated microglial activation, suggesting that maternal-derived microbial signals interfere with the ability of microglia to respond to environmental signals.

Transcriptomic analysis revealed that prenatal stress downregulated IFN-β signaling in both the placenta and fetal brain, while probiotic intervention restored pathway activity. This implies that maternal gut dysbiosis profoundly disrupts immune signaling at the maternal–fetal interface and during offspring neurodevelopment. The placenta is a key mediator of the fetal/maternal interaction by regulating the maternal immune system through signaling pathways while establishing protective mechanisms to shield the fetus from harmful stimuli. During pregnancy, the placenta is continuously exposed to bacterial products through maternal blood circulation. Type I IFNs (IFN-α and IFN-β) are a class of polypeptides capable of inducing an antimicrobial state, modulating innate immune responses, and activating the adaptive immune system.^39^ In the placenta, IFN-β serves as the predominant cytokine. Previous studies have demonstrated that knockout of the IFN-I receptor, which inhibits the IFN-β signaling pathway, leads to a significant increase in placental inflammatory cytokines (e.g., IL-1β) following LPS exposure.^40^ This underscores the critical role of IFN-β signaling in pregnancy. Normal IFN-β signaling is essential for maintaining a healthy pregnancy, as women with dysregulated type I IFN pathways exhibit adverse gestational outcomes, including preeclampsia, preterm birth, and fetal neurodevelopmental impairments.^41-43^ Current findings that connect the maternal gut microbiota with the host immune profile at the maternal‒fetal interface are limited. Recent evidence underscores the critical role of the maternal microbiome in shaping immune responses at the maternal–fetal interface (MFI).^44^ Specifically, germ-free (GF) and antibiotic-treated mice were found to exhibit excessive IFN-γ production linked to fetal resorption, a phenotype that suggests a loss of immune tolerance. Consistent with this concept of microbial immune regulation, our data demonstrate that stress-induced dysbiosis suppresses the placental IFN-β pathway—a protective signal—while probiotic supplementation restores it. Mechanistically, previous studies established that GF mice lack essential tryptophan metabolites, and colonization with the tryptophan-metabolizing commensal *Lactobacillus murinus* effectively reduces MFI IFN-γ levels and prevents fetal resorption. Complementing these findings, our study reveals that prenatal stress favors the production of deleterious tryptophan metabolites (e.g., xanthurenic acid).

Tryptophan serves not only as an essential nutrient but also as a pivotal regulator bridging gastrointestinal physiology and central nervous system function. These regulatory effects are mediated through three major metabolic routes: the indole pathway, the kynurenine (Kyn) pathway, and serotonin (5-HT) synthesis.^45^^,^^46^ The biotransformation of tryptophan yields a diverse array of bioactive metabolites that function as critical signaling molecules, often acting as ligands for the aryl hydrocarbon receptor (AhR).^47^^,^^48^ Among these, the Kyn pathway represents the dominant route of tryptophan catabolism, accounting for approximately 90% of degradation in both immune and epithelial cells.^49^^,^^50^ Kyn and its downstream metabolite kynurenic acid (KA) are known to modulate immune homeostasis, largely through their activity as AhR agonists.^51^ However, research regarding the specific impact of Kyn pathway-derived metabolites at the maternal–fetal interface remains limited. Future work is warranted to elucidate the precise molecular mechanisms by which these specific metabolites modulate placental IFN-β signaling.

Balanced IFN-β signaling is required for normal brain development and function.^52^ Newborn mice from interferon α/β receptor 1 knockout dams exhibited significantly higher proportions of microglia expressing proliferation markers compared to wild-type neonatal microglia.^41^ Both our transcriptomic data and qPCR analyses indicate that prenatal stress downregulates IFN-β-associated pathways in the fetal brain. Notably, IFN-β signaling directly stabilizes the BBB.^52^^,^^53^ Clinically, IFN-β serves as a first-line therapy for multiple sclerosis, partially by reducing BBB leakage to delay disease progression.^54^ Consistent with this protective role, we demonstrated that silencing Ifnar1 compromised BBB integrity, as evidenced by the reduced expression of tight junction proteins. Furthermore, endogenous AhR signaling has been reported to modulate the type I interferon (IFN-I) response,^55^ In the current study, we identified aberrant tryptophan metabolite profiles in the stress group, concomitant with a downregulation of IFN-β signaling in the fetal brain. Crucially, our in vitro experiments demonstrated that 1-MT pretreatment effectively attenuated the downregulation of tight junction proteins induced by *Ifnar1* silencing. Given that tryptophan catabolites are known ligands for AhR, future studies are warranted to confirm whether maternal microbiota-derived tryptophan metabolites influence fetal IFN-β signaling under stress conditions via the AhR pathway.

Substantial research has focused on elucidating the mechanisms governing the transmission of maternal stress to offspring. While stress-induced maternal glucocorticoid release has traditionally been identified as a primary mediator, glucocorticoids alone fail to fully account for the diverse developmental programming effects of psychosocial stress.^56^ Beyond hormones, maternal immune activation and gut microbiota dysbiosis have emerged as critical potential mediators. Rodent studies consistently show that prenatal stress provokes inflammation in the placenta and fetal brain.^11^^,^^57^^,^^58^ However, data regarding systemic maternal inflammation remain inconsistent, with reports of both elevated^59^ and suppressed^60^ inflammatory markers. These heterogeneous findings suggest that relying on a single factor is insufficient to precisely map the complex consequences of prenatal stress on fetal neurodevelopment.

Both clinical and preclinical studies have established that stress and psychiatric disorders are associated with dysbiosis of the maternal and offspring microbiome, as well as an increased risk of neurodevelopmental disorders in the progeny. While clinical research has documented associations between prenatal psychosocial stress and alterations in the microbiome,^61^^,^^62^ the specific taxonomic signatures identified across these cohorts remain inconsistent. This heterogeneity suggests that vertical transmission is unlikely to be the sole mechanism mediating the relationship between maternal stress and offspring behavioral outcomes. Notably, our study utilized cesarean delivery to preclude vertical microbial transmission, thereby unveiling the remote regulatory influence of the maternal microbiota on fetal brain development. In contrast, in a natural birth scenario, offspring would be exposed to the dysbiotic maternal microbiota during delivery. We speculate that this vertical transmission could exacerbate the neurodevelopmental deficits that originate in the fetal stage. Furthermore, previous studies have reported beneficial effects of probiotic mixtures on behavioral phenotypes in maternal separation models,^63^ reinforcing the notion that the microbiota orchestrates neural function throughout the lifespan—from prenatal programming to postnatal maintenance. While direct evidence linking maternal stress to fetal BBB development in humans is limited, our study offers a cerebrovascular perspective on the pathological mechanisms underlying the abnormal behavioral phenotypes observed in adult offspring following prenatal stress.

Our study has several limitations that should be acknowledged. One limitation of this study is that it is based on a mouse model, and further research is needed to extrapolate these findings to humans. Additionally, we analyzed gene expression profiles in whole fetal brains under stress conditions without selectively examining specific cell types or immune cell subsets. Moreover, given the critical role of microglia in embryonic development, future studies are needed to investigate the specific mechanisms by which microglia influence fetal BBB permeability under stress conditions. While our multi-omics analysis reveals a strong association between the gut microbiota, tryptophan metabolites, and IFN-β signaling, we did not employ microbiota-elimination models to definitively prove causality. Future studies utilizing maternal tryptophan supplementation, targeted manipulation of downstream metabolites, or fecal microbiota transplantation (FMT) with specific bacterial taxa are warranted to further validate the causal role of the maternal microbiota in mediating these neuroprotective effects.

## Conclusion

**Figure 8.**
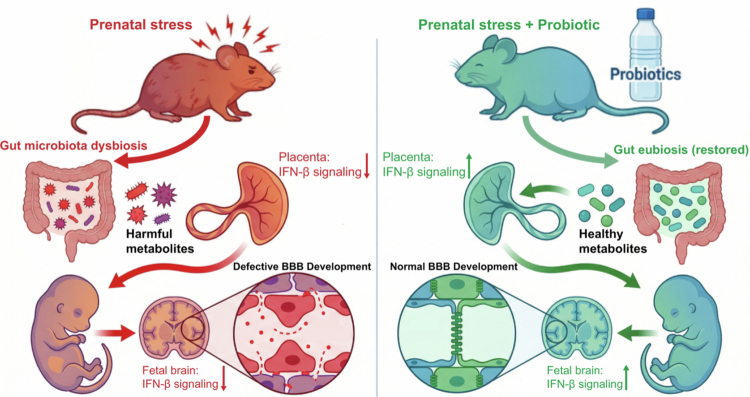
Prenatal stress induces maternal gut microbiota dysbiosis, leading to abnormal tryptophan metabolism and the production of harmful metabolites. These metabolites cross the placenta, where they coincide with downregulated IFN-β signaling in both the placenta and fetal brain. Ultimately, this cascade leads to disrupted endothelial tight junctions and defective BBB development in offspring. Maternal probiotic supplementation during prenatal stress restores the gut microbiota balance and tryptophan metabolism. This normalizes circulating metabolites, restores IFN-β signaling in the placenta and fetal brain, and rescues fetal BBB development by maintaining intact tight junction structures. (Red elements indicate dysfunction; green/blue elements indicate rescue).

This work establishes maternal stress as an inducer of fetal neurodevelopmental defects, with the maternal gut microbiota as a key regulatory node. Crucially, probiotic correction of gestational dysbiosis rescues adverse offspring outcomes. Mechanistically, prenatal stress modulates placenta–brain IFN-β signaling, an effect likely mediated by the maternal microbiota through tryptophan metabolites Figure 8. Our findings elucidate gut–placenta–brain communication under maternal stress and underscore maternal microbial signals as indispensable for neurodevelopmental programming. Overall, a comprehensive understanding of gut bacteria–immune–neural interactions under maternal stress is critical for developing therapeutic strategies for high-risk pregnancies with prenatal depression/anxiety and identifying targets for neurodevelopmental disorder interventions.

## Supplementary Material

Supplementary Material Supplementary_Data.docx

## Data Availability

The 16S rRNA and transcriptomic sequencing datasets are available from the NCBI SRA database with the accession numbers PRJNA1280663 and PRJNA1280015. The metabolomics data have been deposited in the EMBL-EBI MetaboLights database under accession code MTBLS12742 and MTBLS13741.
