# Supplementary material for: Maternal gut microbiota mediates prenatal stress-induced fetal blood‒brain barrier dysfunction
Source: Gut Microbes. 2026 Feb 19;18(1):2631242. doi: 10.1080/19490976.2026.2631242 (PMC12928657; doi:10.1080/19490976.2026.2631242)
Supplement: Supplementary Material — Supplementary_Data.docx [file KGMI_A_2631242_SM0270.docx]

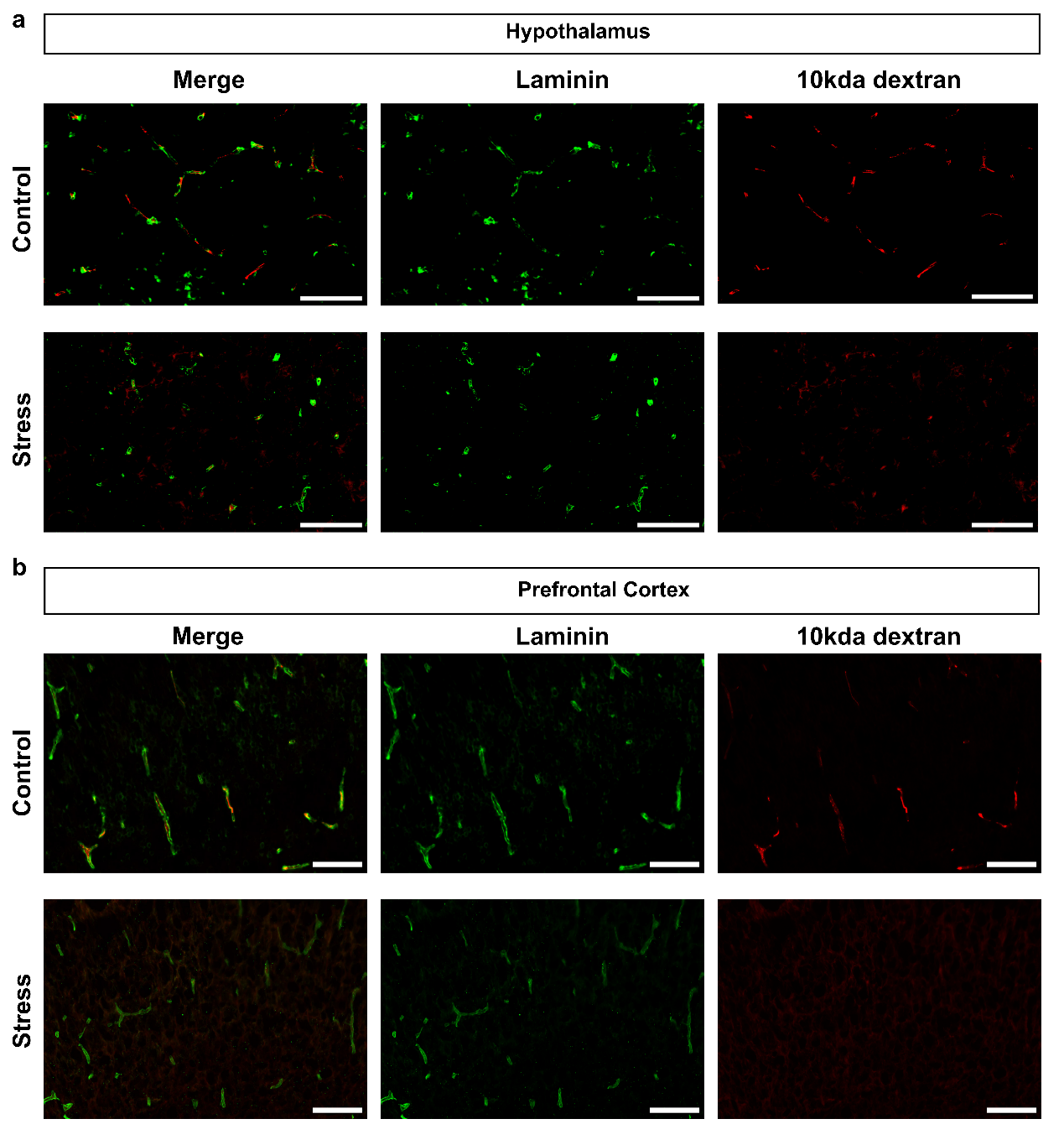


**Figure S1. Prenatal stress induces BBB developmental defects across multiple brain regions in offspring.**

1. Representative fluorescence images of hypothalamus BBB permeability assessment in both offspring groups.
2. Representative fluorescence images of prefrontal cortex BBB permeability assessment in both offspring groups. Scale bar = 100 μm.


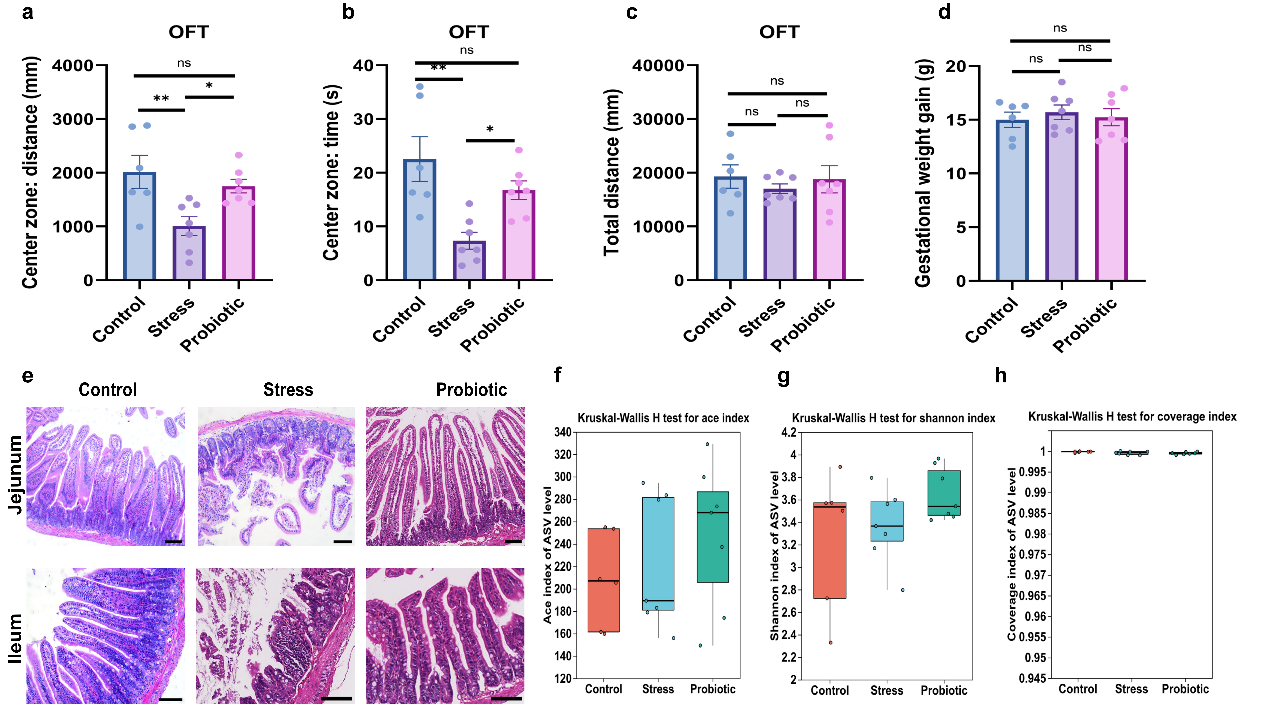


**Figure S2. Maternal probiotic supplementation mitigates stress-induced anxiety-like behaviors and intestinal barrier dysfunction.**

(a-b) Stress dams (n = 7) exhibited significantly reduced distance traveled (***P* = 0.0086 and **P* = 0.0443, respectively) and time spent (***P* = 0.002 and **P* = 0.0434, respectively) in the central zone of the open field compared to controls (n = 6), while probiotic supplementation (n = 7) restored these parameters to control levels.

(c) No significant difference was observed in the total distance traveled among the three maternal groups during the open field test.

(d) No significant difference was observed in gestational weight gain among the three maternal groups.

(e) Representative HE-stained images of jejunum and ileum from the three maternal groups (scale bar = 100 μm).

(f-h) Kruskal-Wallis H test bar plot for ace index (f), shannon index (g) and coverage index (h) from three groups (n = 6 control, 7 stress, 7 probiotic) at the ASV level.

Data were presented as mean ± SEM. **P* < 0.05, ***P* < 0.01; ns, no significant difference. Statistical differences were determined by one-way ANOVA with Tukey’s multiple-comparison test (a-d).


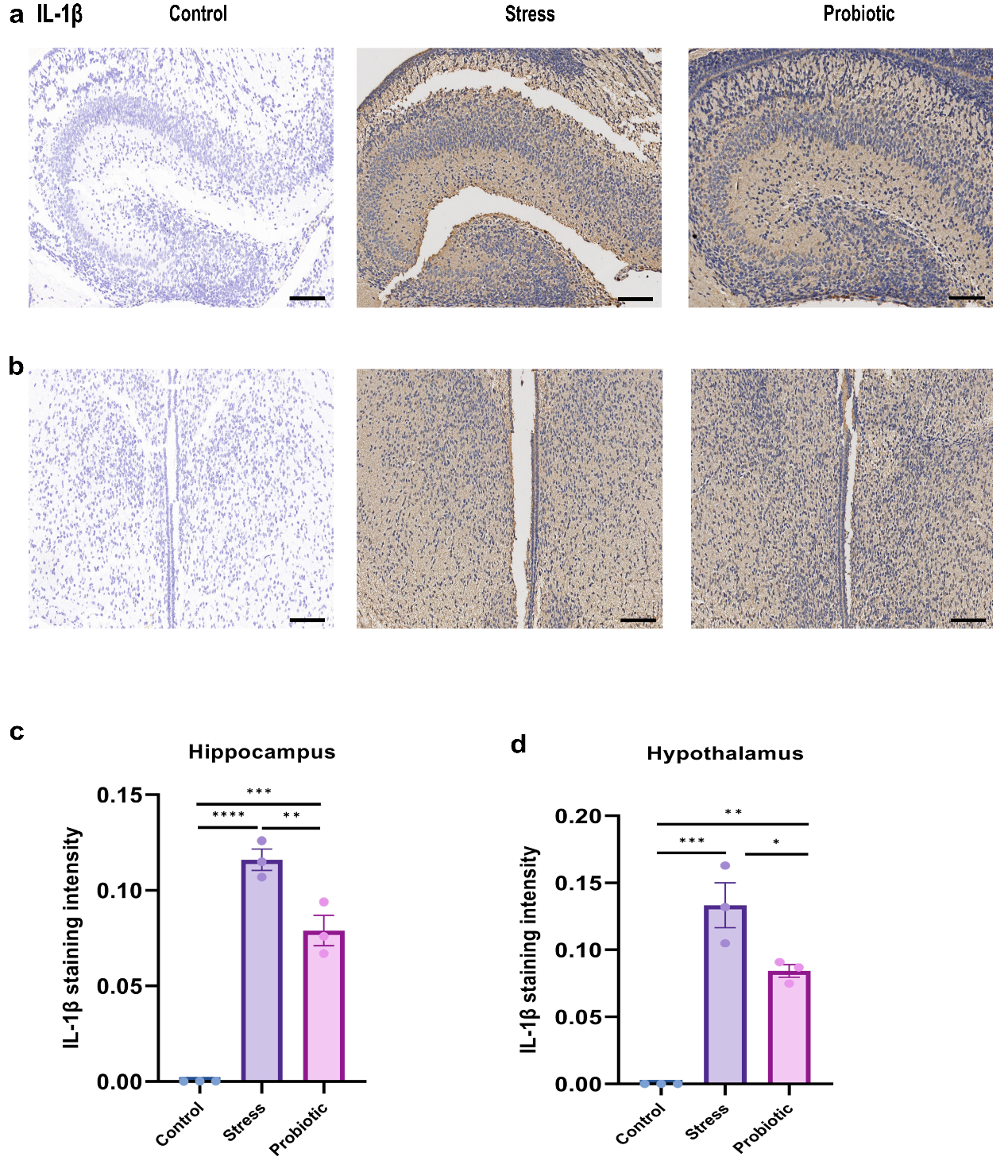


**Figure S3. Prenatal probiotic supplementation improves stress-induced neuroinflammation.**

1. Representative IHC images of IL-1β in fetal hippocampus.
2. Representative IHC images of IL-1β in fetal hypothalamus.

(c-d) MOD quantification revealed elevated IL-1β expression in the hippocampal (*****P* < 0.0001) and hypothalamic (****P* = 0.0002) regions of prenatally stressed offspring, which was reduced following probiotic supplementation (***P* = 0.008 and **P* = 0.0318). Scale bar = 100 μm. Data were presented as mean ± SEM. **P* < 0.05, ***P* < 0.01, ****P* < 0.001 and *****P* < 0.0001; Statistical differences were determined by one-way ANOVA with Tukey’s multiple-comparison test (c-d).


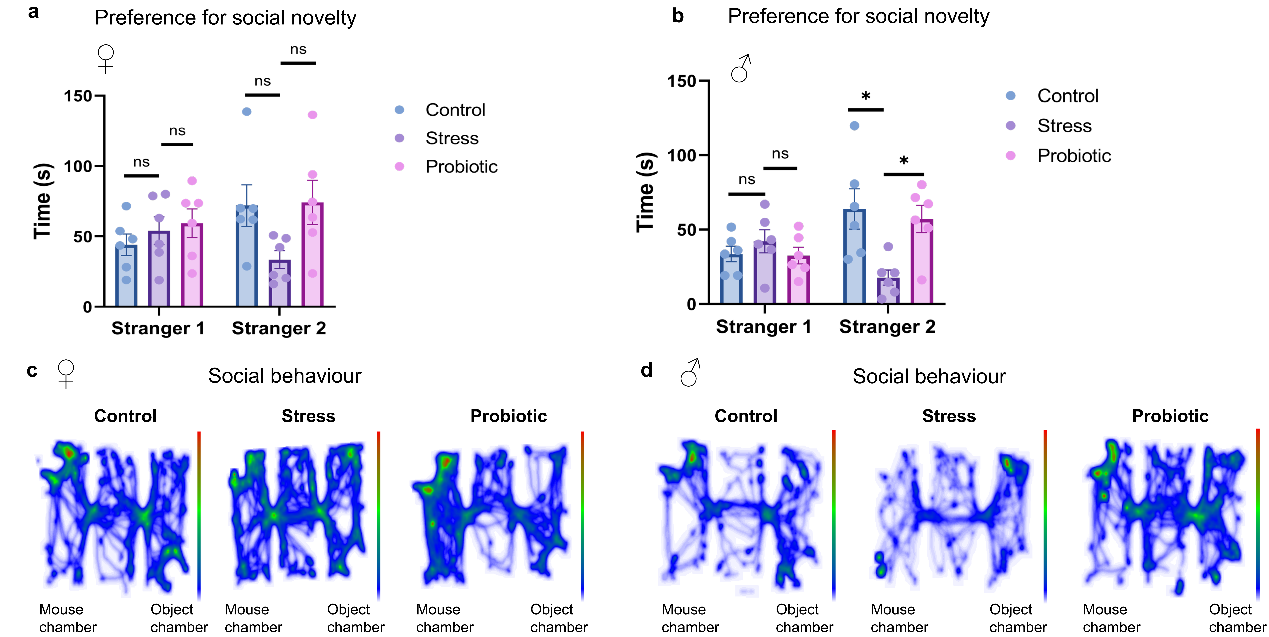


**Figure S4. Prenatal probiotic supplementation improves stress-induced social behavior impairments.**

(a-b) Preference for social novelty of female offspring (a) and male offspring (b, **P* = 0.0130, **P* = 0.0335) from three groups were tested in the three-chambered apparatus (n = 6).

(c-d) The representative motion trajectory density map of female offspring (c) and male offspring (d) from three groups in the three-chamber social approach task. Data were presented as mean ± SEM. **P* < 0.05, ns, no significant difference. Statistical differences were determined by one-way ANOVA with Tukey’s multiple-comparison test (a-b).


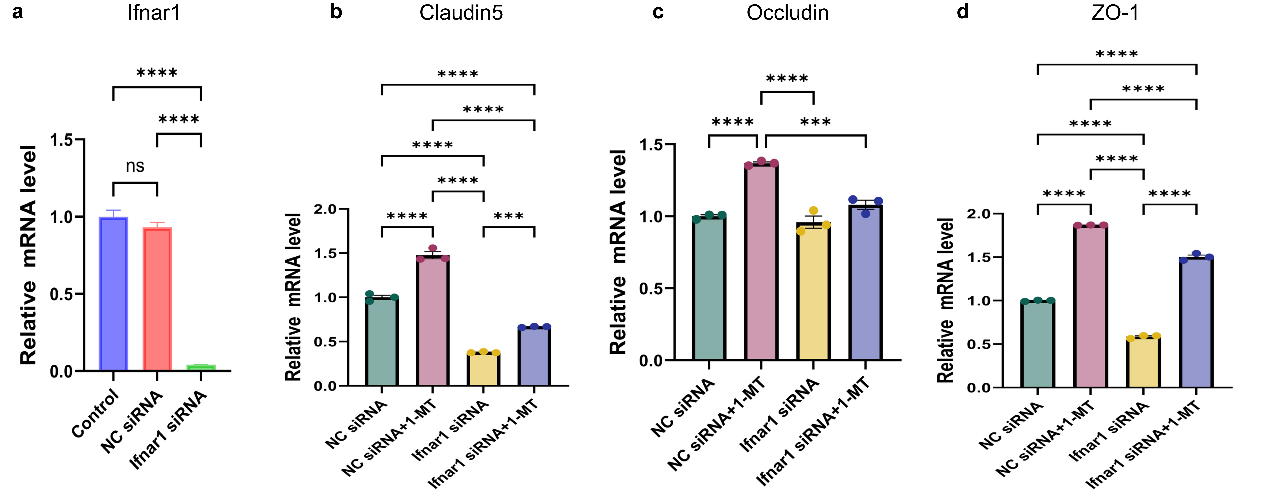


**Figure S5. *Ifnar1* silencing suppresses tight junction protein expression, while 1-MT treatment rescues this defect.**

(a) Relative mRNA expression levels of *Ifnar1* in endothelial cells. Transfection with *Ifnar1*-siRNA significantly reduced *Ifnar1* expression compared to the negative control (NC)-siRNA group (n = 3 samples).

(b-d) Quantitative real-time PCR analysis of tight junction proteins (*Claudin-5*, *Occludin*, and *ZO-1*) in brain microvascular endothelial cells. Cells were transfected with the indicated siRNAs and treated with or without 1-MT (n = 3 samples). 1-MT, 1-methyl-D-tryptophan. Data were presented as mean ± SEM. ****P* < 0.001 and *****P* < 0.0001. Statistical differences were determined by one-way ANOVA with Tukey’s multiple-comparison test (a-d).

Table S1. List of murine expression primers used in this study.

All primers were obtained from BGI Genomics, Shanghai, China.
